# SUN-MKL1 Crosstalk Regulates Nuclear Deformation and Fast Motility of Breast Carcinoma Cells in Fibrillar ECM Microenvironment

**DOI:** 10.3390/cells10061549

**Published:** 2021-06-19

**Authors:** Ved P. Sharma, James Williams, Edison Leung, Joe Sanders, Robert Eddy, James Castracane, Maja H. Oktay, David Entenberg, John S. Condeelis

**Affiliations:** 1 Anatomy and Structural Biology, Albert Einstein College of Medicine, Bronx, NY 10461, USA; edison.leung@gmail.com (E.L.); robert.eddy@einsteinmed.org (R.E.); moktay@montefiore.org (M.H.O.); david.entenberg@einsteinmed.org (D.E.); 2 Gruss Lipper Biophotonics Center, Albert Einstein College of Medicine, Bronx, NY 10461, USA; 3 Colleges of Nanoscale Science and Engineering, SUNY Polytechnic Institute, Albany, NY 12203, USA; jkwilliams333@gmail.com (J.W.); jmsanders905@gmail.com (J.S.); jim.castracane@gmail.com (J.C.); 4 Integrated Imaging Program, Albert Einstein College of Medicine, Bronx, NY 10461, USA; 5 Department of Pathology, Albert Einstein College of Medicine, Bronx, NY 10461, USA; 6 Department of Surgery, Albert Einstein College of Medicine, Bronx, NY 10461, USA

**Keywords:** breast cancer invasion and metastasis, in vivo tumor cell motility, nuclear deformation, LINC complex, SRF-MKL1 signaling

## Abstract

Aligned collagen fibers provide topography for the rapid migration of single tumor cells (streaming migration) to invade the surrounding stroma, move within tumor nests towards blood vessels to intravasate and form distant metastases. Mechanisms of tumor cell motility have been studied extensively in the 2D context, but the mechanistic understanding of rapid single tumor cell motility in the in vivo context is still lacking. Here, we show that streaming tumor cells in vivo use collagen fibers with diameters below 3 µm. Employing 1D migration assays with matching in vivo fiber dimensions, we found a dependence of tumor cell motility on 1D substrate width, with cells moving the fastest and the most persistently on the narrowest 1D fibers (700 nm–2.5 µm). Interestingly, we also observed nuclear deformation in the absence of restricting extracellular matrix pores during high speed carcinoma cell migration in 1D, similar to the nuclear deformation observed in tumor cells in vivo. Further, we found that actomyosin machinery is aligned along the 1D axis and actomyosin contractility synchronously regulates cell motility and nuclear deformation. To further investigate the link between cell speed and nuclear deformation, we focused on the Linker of Nucleoskeleton and Cytoskeleton (LINC) complex proteins and SRF-MKL1 signaling, key regulators of mechanotransduction, actomyosin contractility and actin-based cell motility. Analysis of The Cancer Genome Atlas dataset showed a dramatic decrease in the LINC complex proteins SUN1 and SUN2 in primary tumor compared to the normal tissue. Disruption of LINC complex by SUN1 + 2 KD led to multi-lobular elongated nuclei, increased tumor cell motility and concomitant increase in F-actin, without affecting Lamin proteins. Mechanistically, we found that MKL1, an effector of changes in cellular G-actin to F-actin ratio, is required for increased 1D motility seen in SUN1 + 2 KD cells. Thus, we demonstrate a previously unrecognized crosstalk between SUN proteins and MKL1 transcription factor in modulating nuclear shape and carcinoma cell motility in an in vivo relevant 1D microenvironment.

## 1. Introduction

The tumor microenvironment (TME) plays an essential role in breast cancer invasion and metastasis [1,2,3,4,5]. TME consists of ECM, stromal cells (e.g., cancer associated fibroblasts, adipocytes) and immune cells (e.g., macrophages, neutrophils, etc.) [6,7,8], and drives breast tumor progression through mechanical and chemical cues [1,2,4,8]. The biomechanical properties of tumor ECM (mammographic breast density, ECM stiffness, crosslinking, topography and alignment) play important roles during breast tumor progression. During tumorigenesis, there is an increase in the deposition of matrix proteins, most notably fibrillar collagens, accompanied by extensive matrix remodeling [9,10,11]. Accumulation of collagen I is associated with increased risk of metastasis in breast cancer [12,13,14].

Striking changes in collagen I architecture (topography and alignment) are observed during breast tumor progression. Multiple studies have reported aligned collagen fibers oriented perpendicular to the tumor boundary in human breast tumor tissue sections [15,16] and in xenograft breast tumors in mice [17,18]. In contrast, normal breast stroma contains curly collagen fibrils [11,19,20]. In human samples, collagen fiber alignment correlates with poor disease-specific and disease-free survival [15]. Mammographic breast density, and collagen fiber alignment and cross linking are associated with metastasis and poor prognosis in breast cancer [21,22] and tumor cell dissemination in mouse mammary tumors [23] suggesting an important role for collagen fiber alignment in breast cancer metastasis.

Intravital imaging of mammary tumors has demonstrated that the disseminating tumor cell population moves as single carcinoma cells at high speeds on aligned collagen fibers in vivo [5,24,25,26,27]. Two-dimensional (2D) assays typically used for tumor cell migration studies lack physiological relevance. For example, 2D assays do not capture elongated cellular/nuclear morphology and high-speed migration behaviors observed in vivo [5,28,29,30,31,32,33]. Our goal in this study was to investigate the single carcinoma cell migration behavior on in vivo-like linear collagen fibers. Multiple previous studies have utilized various 1D in vitro assays—micropatterned lines, cylindrical fibers, confined microchannels, and grooved substrates to model cell migration in vivo [28,29,34,35,36,37,38]. These 1D assays capture many of the cellular characteristics observed in vivo, e.g., elongated cellular morphology, rapid cell migration, posterior centrosome position, contractility dependence of migration, and in the case of mammary tumors, alternating linear streams of tumor cells and macrophages [28,30]. Using these 1D and 3D in vitro models of cell motility, fiber alignment was found to be a prominent parameter for cell motility [22,37,39]. However, the relationship of the dimensions of 1D fibers to cell motility phenotype in vivo and reconstitution of this relationship in vitro has been lacking.

Here, we report the development of an in vitro 1D fiber tumor cell motility assay based on the insights obtained from intravital imaging of the modes of cancer cell migration observed in breast tumors and the characterization of mammary tumor ECM fiber dimensions used for this migration in vivo. This 1D fiber tumor cell motility in vitro assay combined with live imaging of single carcinoma cells led to new insights into the regulators of high-speed tumor cell migration in physiological 1D microenvironments. In particular, we found critical roles of ECM fiber diameter and actomyosin contractility constrained to 1D as dominant factors regulating tumor cell motility and nuclear shape changes. In addition, we found and studied a crosstalk between the LINC complex and SRF-MKL1 signaling on the mechanobiology of tumor cells during high speed cell motility in 1D.

## 2. Materials and Methods

### 2.1. Cell Culture, Antibodies, Immunofluorescence

Rat mammary adenocarcinoma, MTLn3 cells were cultured in α-MEM media, supplemented with 5% FBS and antibiotics as described earlier [28,29]. The BAC1.2F5 macrophage cell line [40] was maintained in 10% FBS in α-MEM with 3000 unit/mL CSF-1. Mouse embryonic fibroblasts (MEF) were a kind gift from Louis Hodgson. Cells were transfected with Dendra2-H2B and FAC sorted to generate stable cells. Cells were subsequently maintained in G418 to maintain H2B expression. The following antibodies were used: mouse anti-fibronectin antibody (Abcam, Cambridge, MA, USA, #6328); mouse anti-G-actin (JLA20) [41] and mouse anti-actin (AC-15), SUN1, SUN2, Lamin A/C, Lamin B1, GAPDH, MKL1 (Cell Signaling, Danvers, MA, USA), rabbit anti-FMN2 (GeneTex, Irvine, CA, USA, cat# GTX131161) for Western blot, rabbit anti-FMN2 (Abcam, Cambridge, MA, USA, cat# ab72052) for immunostaining. For immunofluorescence experiments cells were fixed in 4% paraformaldehyde, permeabilized in 0.1% Triton-X, and blocked in 1% BSA + 1% FBS containing PBS before proceeding with primary and secondary antibodies. For G-actin staining, cells were fixed and permeabilized in cold acetone on ice for 5 min according to [41]. Alexa-Fluor 555, 647 Phalloidins were used to label F-actin. CellLight Talin-GFP (Thermo Fisher Scientific, Waltham, MA, USA) was used to label focal adhesions in live cells. Blebbistatin was purchased from Millipore Sigma and used at a concentration of 5 µM. DAPI was used at 1:2000 to label cell nuclei.

### 2.2. Animal Models and Intravital Imaging

All procedures were conducted in accordance with the National Institutes of Health regulation concerning the care and use of experimental animals and with the approval of Albert Einstein College of Medicine Animal Care and Use Committee (IACUC). In vivo second harmonic generation (SHG) imaging for collagen fibers was performed in MTLn3-SCID and PyMT mouse models. Orthotopic tumors were derived from the injection of MTLn3 rat adenocarcinoma cells into SCID mice. PyMT is a transgenic mouse model carrying the mammary tumor virus (MMT-V) polyoma middle T antigen (PyMT). PyMT mice with labeled mammary tumor cells were generated as described previously [42]. Mice were anesthetized with 1–2% isofluorane, skin flap surgeries were performed [43] and animals were placed in a heated chamber maintained at physiologic temperature during the course of imaging. Tumor imaging was performed on a multiphoton Olympus FV1000-MPE microscope or a custom-built multiphoton microscope [44]. Long time-lapse Z-stack imaging was performed by imaging multiple fields of size 512 × 512 μm^2^ for a depth of 100–150 μm beginning at the edge of the tumor. Tumor areas with apparent signs of necrosis were excluded from the analysis.

### 2.3. In Vivo Fiber Diameter Quantification

Analysis of collagen fiber diameters was performed using the images from the SHG channel, making 3D reconstructions of the Z-stack in Imaris and measuring the diameter. For measurements of collagen fibers that were associated with tumor cell motility, 3D reconstructions of the Z-stack time-lapse movies were performed in Imaris and fibers associated with tumor cell motility were measured.

### 2.4. RNAi

AllStars Neg. Control siRNA (1027281) was from Qiagen (Hilden, Germany), rat SUN1 (gene ID:360773, cat# M-080662-01-0005) and rat SUN2 (gene ID:315135, cat# M-081156-01-0005) smartpool siGENOME siRNAs, and MKL1 (gene ID:315151, cat# J-081405-08) ON-TARGETplus siRNA were from Dharmacon (Thermo Fisher Scientific, Waltham, MA, USA). MTLn3 cells were transfected with siRNAs using Oligofectamine (Thermo Fisher Scientific, Waltham, MA, USA) and optimum times for combined maximum KDs were found to be 24 h (SUN1 + SUN2 + MKL1 siRNAs) or 48 h (SUN1 + SUN2 siRNAs).

### 2.5. PVA (Polyvinyl Alcohol) Film Coating

The surfaces of glass substrates were coated with polyvinyl alcohol (PVA) in order to prevent cells from adhering to the 2D glass surface. This protocol was adapted from the literature for use with glass coverslips [38]. Briefly, 12 mm glass coverslips were Piranha Cleaned (3:1, H_2_SO_4_:H_2_O_2_) for 10 min, rinsed 3×, and dried in N_2_. Coverslips were silanized in a container with 3- (amino) propyltrimethoxysilane (APTMS) at 65 °C for 1 h. Substrates were activated using glutaraldehyde for 30 min, rinsed 3×, and dried in N_2_. A 5.6% polyvinyl alcohol (PVA, Sigma-Aldrich, St. Louis, MO, USA) solution was solubilized at 90 °C, sonicated, and filtered through a 0.2 μm syringe filter. An 11.24% 2N HCl was added to the filtered solution. In total, 65 μL of the PVA solution was added to each coverslip for 5 min followed by spincoating at 7000 RPM for 40 s. The coated coverslips were cured overnight at 4 °C. A ring of polydimethylsiloxane (PDMS) was placed around the coverslip boundary in order to suspend the electrospun fibers over the PVA surface.

### 2.6. Electrospun Fibers

One-dimensional aligned microfibers were created from a 21% (*w*/*w*) poly lactic-co-glycolic acid, PLGA (LACTEL) solution dissolved in hexafluoroisopropanol (HFIP, Krackeler) with 1% NaCl (Sigma Aldrich, St. Louis, MO, USA) and stirred overnight. The solution was fed into a 3 mL syringe, the flow rate was controlled by a syringe pump, and the high voltage was applied using a high voltage power supply. The aligned microfibers were attracted to two grounded parallel collector plates consisting of cleaved silicon. A PVA coated glass coverslip was lifted between the parallel collector plates to transfer the electrospun fibers. The collector plates were 15 cm from the needle tip. The flow rate was set to 16 µL/min and the voltage was set to 12 kV. The fibers were allowed to collect between the parallel plates for 6 s before the pump was stopped and the fibers were collected.

### 2.7. ECM Coating of Fibers

The cover glass from a MatTek dish (10 mm well) was removed and the electrospun fiber containing 12 mm cover glass was attached to the MatTek dish using silicone glue. Alexa-568 labeled fibronectin (final conc = 10 ug/mL) or FITC labeled collagen I (final conc = 40 ug/mL) was added to the central well to completely cover the fibers. After 2 h, fibers were washed 2× to remove the excess fluorescent ECM and dishes were stored in dark at 4 °C. Labeled fibers were used within 1–2 days.

### 2.8. Fibronectin-Coated Micro-Patterned 1D Lines

Custom made fibronectin-coated micro-patterned 1D lines and 2D areas (cat #1D2D EDDY) were purchased from CYTOO Inc. In addition, CYTOO chips Motility (CYTOO Inc., 38,040 Grenoble, France) was used as described [28]. Dimensions of 1D lines were evaluated with AFM. All AFM measurements were taken with the Bruker Bioscope Catalyst. To understand the nanoscale topography of these lines, the chips were measured using ScanAsyst probes in both contact mode and PF-QNM. Different methods were used to ensure that the topography was not an artifact of the large differences in the adhesive properties of the lines. These measurements were performed in air, PBS, and 10× PBS, which would reduce any charge interactions between the probe and the substrate. In every condition, the scans of the surface revealed that the fibronectin lines were a mean thickness of 2.5 µm and a depth of 3.5 nm (Figure S1).

### 2.9. Scanning Electron Microscopy

Scanning electron microscopy was performed using a Leo1550 scanning electron microscope. This technique was used to measure microfibers to ensure that they were of the desired diameter. Fibers were coated by bombardment with gold-palladium particles in an argon gas environment. The chamber was pumped down to 50 mTorr and subsequently flooded with argon gas. The chamber was again pumped down to 50 mTorr and then coated with the gold-palladium particles. This process was used to reduce the charging effect of the specimen because of the high electrical conductivity of the gold-palladium coating. The in lens detector was used with the 20 µm aperture at 5 kV. The sizes of fibers were determined using Image J by measuring 10–20 fields in each preparation.

### 2.10. Fibronectin Staining of ECM in Tumor Sections

MTLn3 orthotopic tumors grown in SCID mice were removed and immediately frozen in liquid nitrogen. Tumor sections were cut for immunostaining. Tissue sections were blocked in donkey serum for 45 min and mouse anti-fibronectin antibody (Abcam, Cambridge, MA, USA, cat#6328) was added at 1:100 dilution for 1 h. Sections were washed 3× in PBS and then incubated with a donkey anti-mouse Alexa 555 secondary antibody at 1:1000 dilution (Thermo Fisher Scientific, Waltham, MA, USA) for 1 h. Secondary antibody was washed 3× with PBS. Images were obtained on a custom built multiphoton microscope described previously [44].

### 2.11. Live-Cell Microscopy

Time lapse images were acquired on a wide-field DeltaVision microscope equipped with a high precision X and Y NanoMotion III stage for capturing images at multiple positions and a Photometrics CoolSnap HQ2 CCD camera (Applied Precision, GE Healthcare, Chicago, IL, USA). Imaging was done at 37 °C with a 20× air objective, NA = 0.4. Up to 10–15 different fields along the linear dimension were imaged in the phase contrast and FITC channels every 2 min for up to 10–12 h. For tumor cells and BACs co-migration experiment, 25,000 MTLn3 cells were plated on fibronectin-coated 1D fibers overnight. MTLn3 cells were imaged alone for 2 h, followed by addition of 100,000 BACs to the imaging chamber on the microscope stage and imaging was continued for the next 6–8 h. For tumor cell migration parameter calculations, we only analyzed tumor cells moving on a single fiber in the areas where fibers do not cross each other.

### 2.12. Single Tumor Cell Tracking in 1D and 2D

A custom ImageJ/Fiji macro (included with this article) was used to track single cells based on nucleus centroid tracking in GFP-H2B time-lapse movies (Figure S3, Movie S4). Only single nondividing cells were tracked. The macro calculates aspect ratio as well as raw centroid values, which were used to calculate cell speed and persistence values as described earlier [28]. Briefly, instantaneous cell speed was calculated every 2 min by dividing the distance of nucleus centroid traveled by the time, 2 min. Average cell speed was calculated by taking the average of all the instantaneous speeds during the whole cell tracking period. Instantaneous persistence was calculated every 2 min by the formula: speed/[1 + (100/360) × angle], where angle is the directional change in degrees [28,45]. For 1D cell motility, angle = 0 or 180 depending on whether the cell continues moving in the same direction or rapidly switches the direction. Average persistence was calculated by averaging the instantaneous persistence values. For cellular aspect ratio calculations, cell outlines were manually traced frame-by-frame in time-lapse movies and aspect ratio was measured in ImageJ/Fiji.

### 2.13. F-Actin Stress Fiber Imaging and Quantification

Cells were plated on Alexa-568 conjugated fibronectin coated 2 µm fibers or CYTOO fibronectin 650 motility chip containing fibronectin 650-coated 2.5, 5, 10, and 20 um lines as well as 2D areas. At room temperature, cells were fixed in 4% PFA for 20 min, permeabilized with 0.1% Triton-X for 5 min and incubated with 633-phalloidin (for cells on 2 µm fibers) or 546-phalloidin (for cells in CYTOO fibronectin 650 motility chip) for 20 min to fluorescently label stress fibers. Fixed cells were imaged on DeltaVision microscope equipped with a high precision X and Y NanoMotion III stage for capturing images at multiple positions and a Photometrics CoolSnap HQ2 CCD camera (Applied Precision, GE Healthcare, Chicago, IL, USA). Cells were imaged with a 60× oil objective in fibronectin and phalloidin channels. For F-actin stress fiber angle quantification, images in the phalloidin channel were analyzed in ImageJ/Fiji by manually drawing lines along the F-actin stress fibers and measuring the angle of those fibers with respect to the 1D axis in the fibronectin channel. Histogram plot of percentage of F-actin fibers in each case (2 µm fiber, 2.5 µm line, etc.) was generated in Excel by binning all fiber angles in each case into 0–10, 10–20, 20–30, and so on, angle bins.

### 2.14. Live Imaging and Quantification of Lifeact Cells

TagRFP-Lifeact was purchased from Addgene, Watertown, MA, USA (cat # 54586). MTLn3 cells stably expressing TagRFP-Lifeact were generated by transiently transfecting with the plasmid [46] and selecting with 0.8 mg/mL Geneticin. Cells were further FAC sorted to collect cells expressing medium levels of TagRFP-Lifeact. Stable TagRFP-Lifeact expressing cells were plated on fibronectin-coated fibers overnight. The next day, cells were imaged live on DeltaVision microscope using a 20× objective every 2 min. Half-way through the imaging, 5 µM Blebbistatin (final concentration) was added in the dish on the microscope stage and time-lapse imaging was continued. Lifeact intensity was quantified by drawing lines of width 4 pixels (~2.5 µm) along the 1D axis covering the length of the same cell before and after blebbistatin treatment.

### 2.15. Analysis of SUN1 and SUN2 Expression in Cancer Patients

Expression of SUN1 and SUN2 in primary tumor vs. normal breast tissue was performed on TCGA dataset for breast invasive carcinoma using UALCAN, http://ualcan.path.uab.edu/analysis.html [47], accessed on 2 November 2018. Analysis of SUN1 and SUN2 expression in tumor vs. normal tissue across tumor types was performed using GEPIA, http://gepia.cancer-pku.cn [48], accessed on 2 November 2018.

### 2.16. Statistical Analysis

All experiments were performed for at least 3 independent biological replicates. Statistical significance was calculated using unpaired, two-tailed Student’s *t*-test and statistical significance was defined as *p* value < 0.05. The * indicates *p* value < 0.05; ** indicates *p* value < 0.01; *** indicates *p* value < 0.001 and error bars represent the SEM.

## 3. Results

### 3.1. Breast Carcinoma Cells Show Fast Motility Primarily on Small Diameter ECM Fibers In Vivo

**Figure 1:**
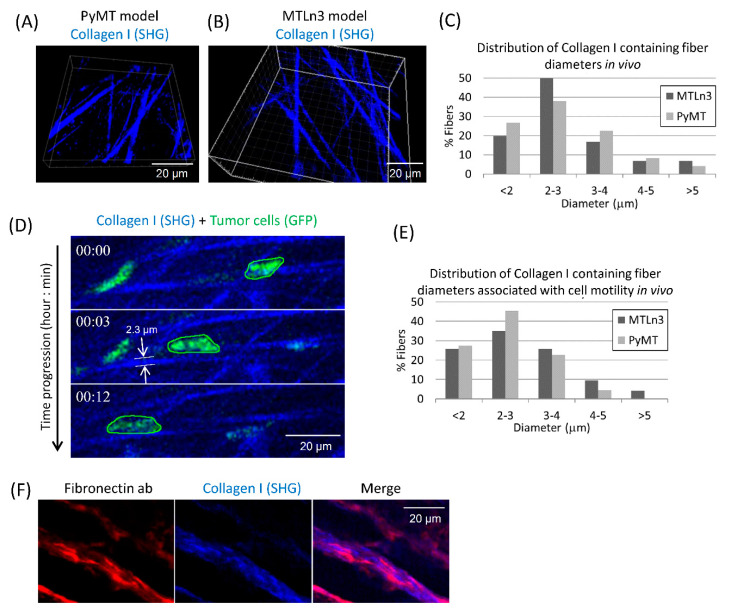
In vivo characterization of collagen fiber diameter and composition in mammary tumors. (**A**,**B**) Stills from the 3D reconstruction movies of collagen fibers in vivo using second harmonic generation (SHG) intravital imaging in two mice models—PyMT (**A**) and MTLn3 (**B**). (**C**) Histogram of collagen fiber diameters (µm) in PyMT and MTLn3 models. N = 162 (MTLn3) and 71 (PyMT) fibers analyzed. (**D**) A representative time-lapse sequence of a tumor cell (green) moving on linear collagen I containing ECM fiber (blue, ~2.3 µm diameter, shown in the middle panel) in vivo in PyMT mouse. (**E**) Histogram of tumor cell motility-associated collagen fiber diameters (µm) in PyMT and MTLn3 models. N = 74 (MTLn3) and 44 (PyMT) fibers analyzed. (**F**) Immunofluorescence staining of MTLn3 tumor sections with fibronectin antibody (red, left panel) with SHG signal for collagen fibers (blue, middle panel) demonstrates that ECM fibers in vivo are composed of both fibronectin and collagen I.

In order to reconstitute in vitro tumor cell motility with high fidelity to the in vivo tumor cell motility phenotype, we first determined the in vivo tumor ECM architecture that is preferred by tumor cells during their migration. The MTLn3 and PyMT breast cancer mouse models are two of the best characterized mouse models of tumor cell invasion, motility and metastasis [30,49,50]. In these breast cancer models it is well established that tumor cells migrate on linear fibrillary ECM fibers [5,11,16,28,30,50]. Using these mammary tumor models, we imaged the fibrillar collagen I associated with tumor cell motility in vivo using second harmonic generation (SHG) intravital microscopy (Figure 1A,B, Movie S1). We made 3D reconstructions from the z-stack movies of collagen I containing ECM fibers, and as expected, found that the collagen I containing fibers had a round topography (Movie S1). We analyzed 3D reconstruction movies for collagen I containing fiber diameters and found that the most common fiber diameter, in both MTLn3 and PyMT models, was in the 2–3 µm range with the majority being <3 µm in diameter (Figure 1C). Consistent with previous literature [5,11,16,28,30,50], we found that tumor cells moved along linear collagen I containing ECM fibers (Figure 1D). More importantly, we found that tumor cells in vivo prefer to move on fibers of <3 µm in diameter (Figure 1E).

Next, we determined the ECM composition of these in vivo fibers. We stained frozen tumor sections with fibronectin antibody and found that ECM fibers are composed of both collagen I and fibronectin (Figure 1F). We also checked for other ECM molecules, such as laminin and collagen IV, but did not see any staining in tissue sections (data not shown).

### 3.2. Fabrication and Characterization of In Vitro 1D Fibers

**Figure 2:**
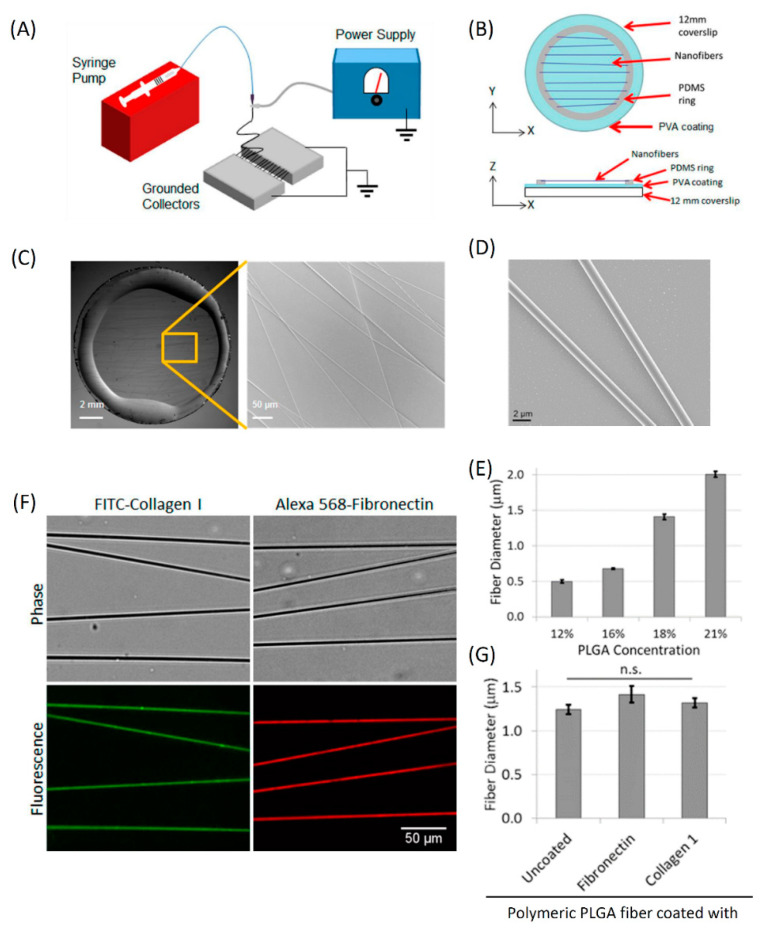
The in vitro 1D fiber assay setup: fabrication of PLGA fibers, their size characterization and ECM coating. (**A**) Schematic of electro-spinning technique setup for the 1D fiber generation. (**B**) A cartoon depicting the components of 1D fiber assay setup in both X-Y and X-Z views. Fibers are glued at two ends to a PDMS ring, which keeps fibers suspended in air above the PVA-coated 2D surface (cyan). (**C**) Images of 1D fibers deposited on PDMS glue ring, which keeps fibers suspended in air. Individual fibers can be seen in the zoomed view. (**D**) Scanning electron microscopy high-resolution image of 1D fibers showing cylindrical morphology of individual fibers. (**E**) Quantification of PLGA concentration vs. 1D fiber diameter using scanning electron microscopy images of fibers spun at different PLGA concentrations. Data plotted as mean ± SEM, *n* = 6, 10, 9, 23 fibers analyzed for 12%, 16%, 18%, and 21% PLGA concentrations. (**F**) Images of 1D fibers coated with different ECM molecules (collagen I or fibronectin), showing a uniform and selective coating of 1D fibers with no ECM deposition underneath the fibers on the 2D surface. (**G**) Quantification of 1D fiber diameter with and without different ECM coatings. Data plotted as mean ± SEM, *n* = 9 fibers for each case.

Based on our observations of single breast tumor cell migration on collagen fibers in vivo [5,28,30] and the fiber diameter characterization we performed above, we set out to create artificial linear fibers matching the in vivo ECM fiber diameter and composition. We used electro-spinning technology to generate polymeric fibers made from poly lactic-co-glycolic acid (PLGA) (Figure 2A). Fibers were electro-spun and suspended over a glass coverslip by attaching them to the PDMS glue at two ends (Figure 2B). To avoid cell attachment in the 2D area, the glass coverslip was coated with PVA [38] to prevent spreading of tumor cells on the PVA surface (Figure S1). We performed bright-field microscopy to visualize the fibers and observed single linear fibers (Figure 2C). To visualize PLGA polymeric fibers at higher resolution, we imaged them using scanning electron microscopy and quantified the fiber diameters (Figure 2D,E). We found that we can generate PLGA polymeric fibers with diameters in the nanometer to micrometer range by changing PLGA concentration, with 21% PLGA generating the 2 µm fibers.

Next, ECM (collagen I or fibronectin) coating conditions were optimized to obtain a uniform ECM coating on PLGA polymeric fibers (Figure 2F). To check the effect of ECM coating on fiber thickness, we performed atomic force microscopy (AFM) on uncoated and ECM coated PLGA polymeric fibers and found no significant difference in fiber diameter with or without the ECM coating (Figure 2G).

### 3.3. Dimensionally Matched Physiological 1D Substrates Mimic In Vivo Fast Tumor Cell Motility

**Figure 3:**
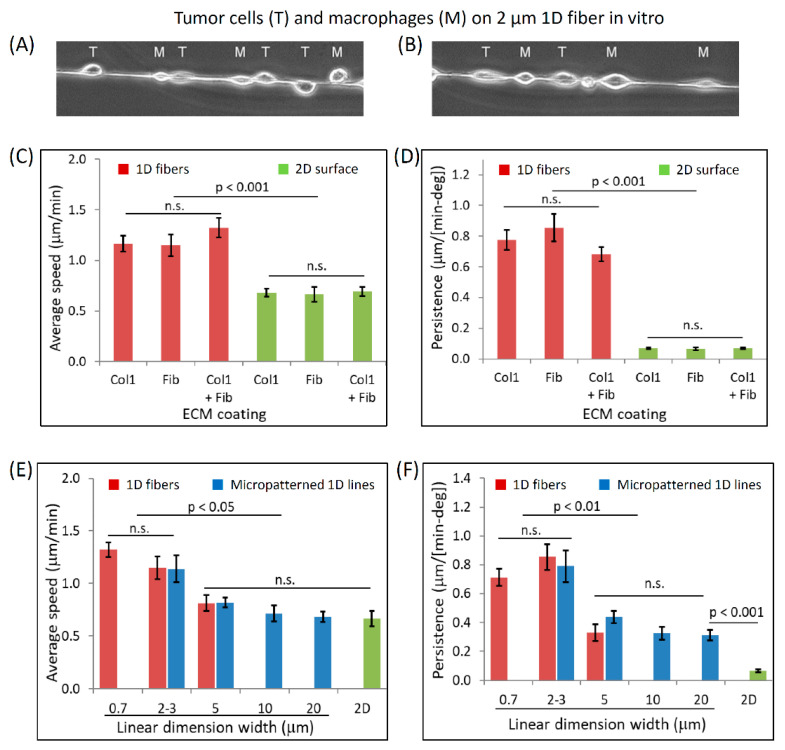
Tumor cells exhibit high-speed migration on thin ECM-coated 1D fibers. (**A**,**B**) Assembly of streams composed of alternating cell types is a cell autonomous property on 1D fibers. Two examples of tumor cells (MTLn3, labeled T) and macrophages (BAC1.2F5, labeled M) showing alternating streaming pattern on 1D fibers. (**C**,**D**) Quantification of average cell speed (**C**) and persistence (**D**) of tumor cells migrating on 2 µm fibers vs. on 2D surface coated with different ECM molecules (Collagen I, Fibronectin or Collagen + Fibronectin). Red and green bars represent measurements made on ECM coated (Collagen I, Fibronectin or Collagen I + Fibronectin) 2 µm fibers and on 2D surface, respectively. Data plotted as mean ± SEM; *n* = 25, 18, 20, 23, 8, and 22 cells for 1D-Col1, 1D-Fib, 1D-Col1 + Fib, 2D−Col1, 2D−Fib, and 2D−Col1 + Fib case, respectively. (**E**,**F**) Quantifications of average cell speed (**E**) and persistence (**F**) of tumor cells migrating on different thickness 1D fibers (0.7, 2, and 5 µm), micro-patterned 1D lines (2.5, 5, 10, and 20 µm) or 2D surface. Red, blue and green bars represent measurements made on fibronectin coated 1D fibers, 1D micro-patterned lines, and 2D surface, respectively. Data plotted as mean ± SEM; *n* = 21, 18, and 19 cells for 0.7, 2–3, and 5 µm 1D fibers respectively; *n* = 20, 17, 16, and 20 cells for micropatterned lines of width 2.5, 5, 10, and 20 µm, respectively; *n* = 8 cells for the 2D case.

To evaluate the in vivo relevance of our 1D fiber assay, we plated mammary carcinoma cells (MTLn3) on ECM coated PLGA fibers and performed live imaging (Movie S2). Cells displayed elongated morphology while moving on fibers, similar to what is seen in vivo. Another characteristic feature of carcinoma cell migration in vivo is the formation of the multicellular streaming pattern containing alternating tumor cells and macrophages [28,30]. Tumor cells and macrophages, when plated together on the ECM-coated fibers in vitro, arranged themselves in the alternating streaming pattern seen in vivo (Figure 3A,B, Movie S3). Encouraged by the in vivo relevance of our in vitro 1D fiber assay, next we carried out a comprehensive investigation of single tumor cell motility characteristics (speed and persistence) on fibers of different diameters and ECM coatings, and compared it with motility parameters of the same cells on a 2D surface of the same ECM (Figure S3, Movie S4). We found that, on 1D fibers tumor cells move twice as fast and with 10 times higher persistence than in 2D (speed: 1.2 vs. 0.6 µm/min, and persistence: 0.75 vs. 0.07 µm/min-deg in 1D vs. 2D, respectively) (Figure 3). Interestingly, in both 1D and 2D, we saw similar cell speed and persistence values (Figure 3C,D) whether the surface (1D or 2D) on which cells are moving was coated with fibronectin alone or collagen 1 alone or with fibronectin + collagen 1. Therefore, we performed all subsequent experiments on fibronectin-coated 1D or 2D surfaces for the sake of simplicity.

Next, we examined the effect of varying fiber diameter on tumor cell motility. On smaller diameter fibers (700 nm), tumor cells moved at the same speed and persistence as on 2 µm fibers (Figure 3E,F). On thicker fibers (5 µm), there was an approximately 30% decrease in cell speed and 60% decrease in persistence, indicating that thicker fibers are not optimum for efficient tumor cell migration. Due to high polymer viscosity at higher PLGA concentrations, we were unable to electrospin fibers >5 µm in diameter. Therefore, we resorted to using fibronectin-coated micro-patterned 1D lines to study tumor cell motility on >5 µm 1D lines. First, we compared tumor cell motility parameters on 2.5 and 5 µm thick 1D lines versus 2 and 5 µm thick fibers. We found that tumor cells move at the same speed and persistence on 2.5 and 5 µm 1D lines as they do on 2 and 5 µm fibers, respectively (Figure 3E,F), indicating that micro-patterned 1D lines and 1D fibers can be used interchangeably for tumor cell motility studies. As we increased the micro-patterned line width to 10 and 20 µm, cell speed plateaued out and approached the 2D tumor cell speed (~0.6 µm/min). Tumor cell persistence also showed a decreasing trend with increasing 1D line width, although it never reached the 2D persistence value even at 20 µm line width, possibly because the boundary of 1D line restricts cell movement perpendicular to the line axis and contributes toward higher cell persistence, whereas in 2D, cells are free to move in any direction.

It is important to note that (1) cell motility (both persistence and speed) on all fibers of ≤3 µm diameter is not significantly different between diameters within this group (Figure 3E,F and (2) the coating does not affect fiber diameter (Figure 2G).

### 3.4. Actomyosin Contractility Regulates F-Actin Alignment along 1D Axis and High-Speed Tumor Cell Motility in 1D

**Figure 4:**
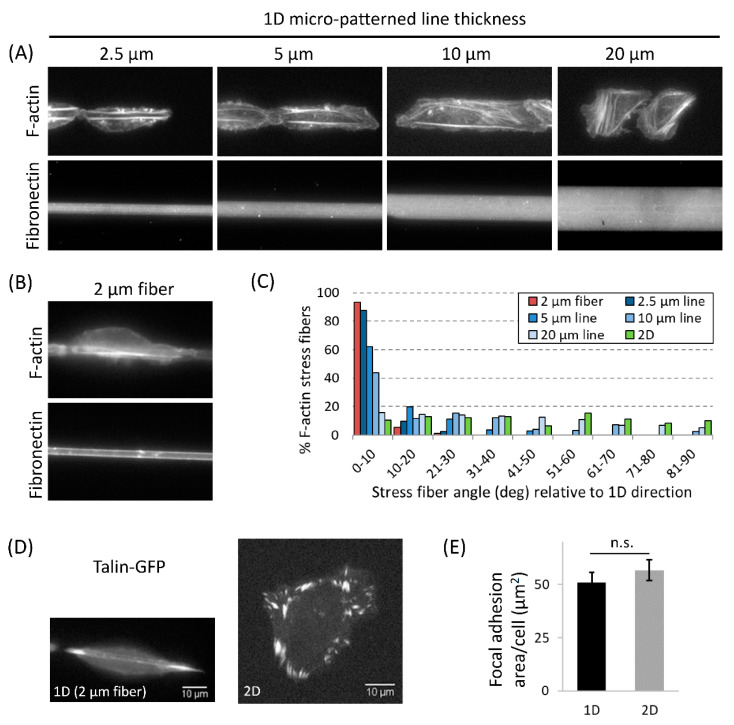
Enhanced alignment of F-actin stress fibers and focal adhesions in tumor cells moving in 1D. (**A**) F-actin staining in tumor cells moving on different thickness micro-patterned 1D lines (2.5, 5, 10, and 20 µm). (**B**) F-actin staining in tumor cell moving on fibronectin-coated 2 µm polymeric fiber. (**C**) Quantification of the proportion of F-actin stress fibers (from all cells in each condition) aligned at the specific angle with respect to the 1D axis shown as histogram plot for each condition. Angles 0 and 90 degrees represent the direction parallel and perpendicular to the 1D axis, respectively. *n* = 90, 81, 106, 123, 158, and 170 F-actin stress fibers analyzed in 12, 14, 12, 13, 13, 12 cells on 2 µm fiber, 2.5 µm line, 5 µm line, 10 µm line, 20 µm line and in 2D, respectively. (**D**) Focal adhesion (CellLight Talin-GFP) distribution in tumor cells shows aligned focal adhesions in cells moving in 1D (2 µm fibers) vs. randomly oriented focal adhesions in cells moving in 2D. (**E**) Quantification of total focal adhesion area/cell in cells moving in 1D (2 µm fibronectin-coated polymeric fibers) and 2D (fibronectin-coated). Data plotted as mean ± SEM, *n* = 23 and 20 cells for the 1D and 2D conditions, respectively.

Since actin is the dominant cytoskeleton component contributing toward cell migration [24,51], we investigated F-actin distribution in tumor cells plated on different width micro-patterned 1D lines and fibers. F-actin stress fibers were primarily oriented along the 1D axis in cells moving on 2 µm fibers or 2.5 µm lines (Figure 4A,B). With increasing 1D line width (5, 10, and 20 µm), F-actin stress fibers in cells gradually lost their alignment along the 1D axis and became oriented in all possible directions, approaching the 2D stress fiber alignment distribution (Figure 4A–C). Since higher tumor cell motility parameters correlate with higher F-actin stress fiber alignment along the 1D axis, we hypothesize that the aligned F-actin on 2–3 µm 1D lines and fibers helps focus protrusive forces along the 1D axis, leading to high carcinoma cell speed and persistence. In support of this hypothesis, we also found the alignment of focal adhesions along the 1D axis in carcinoma cells moving on 2 µm fibers, compared to numerous and often smaller focal adhesions typically seen in cells moving on 2D (Figure 4D, Movie S5). It is interesting to note that the total focal adhesion area does not change in cells moving in 1D vs. 2D (Figure 4E). Rather randomly oriented focal adhesions seen in cells in 2D get aligned along the 1D axis in cells moving in 1D.

**Figure 5:**
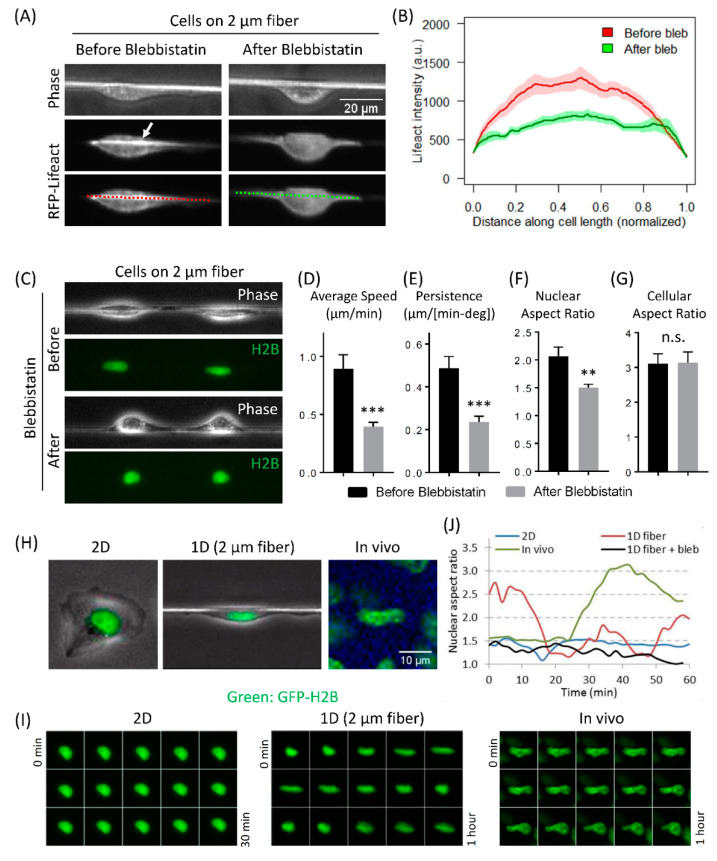
Actomyosin contractility regulates cell-intrinsic nuclear deformation and tumor cell motility in 1D. (**A**) Phase and RFP channel images of RFP-Lifeact (a marker for F-actin) expressing tumor cells migrating on fibronectin-coated 2 µm polymeric fiber before and after blebbistatin treatment. White arrow indicates a linear bundle of F-actin stress fibers. Red and green dotted lines in the bottom image panels indicate the lines along which the Lifeact intensity measurements were made and quantified in Figure 5B. (**B**) Quantification of RFP-Lifeact intensity along the cell length (marked with red and green dotted lines in Figure 5A) migrating on fibronectin-coated 2 µm polymeric fiber before and after blebbistatin treatment. Data plotted as line plot with solid lines showing the mean values and shaded areas representing SEM error bars around the mean, *n* = 13 cells. (**C**) Frames from time-lapse Movie S6, showing phase and GFP-H2B channels for MTLn3 cells moving on fibronectin-coated 2 µm polymeric fibers. Mid-way during the movie, cells were treated with 5 µM blebbistatin to inhibit actomyosin contractility. Note the nuclear morphology change from elongated to round after the blebbistatin treatment. (**D**–**G**) Quantifications of average cell speed (**D**), persistence (**E**), nuclear aspect ratio (**F**), and cellular aspect ratio (**G**) of tumor cells migrating on fibronectin-coated 2 µm polymeric fibers, before and after blebbistatin treatment. Data plotted as mean ± SEM, *n* = 13 cells. ** *p*-value < 0.01, *** *p* value < 0.001, n.s. not significant. (**H**) Still images from Movies S7–S9 of GFP-H2B MTLn3 cells moving in 2D, 1D (2 µm fibers), and in vivo, showing rounder nuclear morphology in 2D vs. elongated nuclear morphology in 1D and in vivo. (**I**) Time-lapse images of nucleus (GFP-H2B) shape dynamics, show static rounder nuclear morphology in cells moving in 2D, vs. dramatic nuclear shape changes observed in cell moving in 1D and in vivo. Time-lapse intervals are 2, 4, and 4 min for 2D, 1D (2 µm fibers), and in vivo, respectively. (**J**) Plots of nuclear aspect ratio change over time in cells moving on 2D substrate, in vivo, and in 1D. Note the actomyosin dependence of nuclear shape change in 1D.

To test the role of aligned F-actin in generating protrusive forces in 1D, we inhibited actomyosin contractility in cells moving in 1D with 5 µM blebbistatin. Using Lifeact as a marker for F-actin in live carcinoma cells, first, we checked F-actin levels in cells before and after the inhibition of actomyosin contractility, and found that there was a sharp decrease in F-actin stress fiber intensity along the 1D axis after actomyosin contractility inhibition (Figure 5A,B). This decrease in F-actin intensity after actomyosin contractility inhibition correlated with dramatic decreases in cell speed and persistence (Figure 5C–E).

### 3.5. Fast Tumor Cell Motility and Nuclear Shape Changes Are Linked Together in 1D and In Vivo, but Not in 2D

During our studies on the effect of blebbistatin on cell migration in 1D, we also observed that inhibiting actomyosin contractility led to a rounder nuclear shape compared to the elongated shape observed in untreated cells (Figure 5C, Movie S6). We quantified nuclear aspect ratio and found a significant decrease in nuclear aspect ratio after inhibition of actomyosin contractility (Figure 5F). We also checked the effect of blebbistatin treatment on cellular aspect ratio and interestingly found that blebbistatin does not change cellular morphology (Figure 5G), indicating that nuclear and cellular morphological shape changes are not coupled in cells moving in 1D. These results suggest that nuclear shape may be intimately linked to the motility potential of tumor cells in 1D, i.e., more elongated the nuclear shape, the higher the motility parameters and vice versa.

To check the link between nuclear shape and cell migration, we investigated the nuclear shape of carcinoma cells in 2D, 1D and in vivo. Consistent with high carcinoma cell motility in 1D and in vivo, as described above, we found that the nucleus is elongated (i.e., high nuclear aspect ratio) in cells moving in 1D in vitro and in vivo (Figure 5H). In contrast, the nucleus in cells moving in 2D was found to be round (i.e., low nuclear aspect ratio) (Figure 5H). When we examined nuclear morphology more closely in live movies of GFP-H2B expressing carcinoma cells moving in 1D, we found that nuclear shape showed dynamic elongation and contraction cycles (Figure 5I, Movie S7), compared to static rounder nuclear morphology in cells moving on 2D surface (Figure 5I, Movie S8). Interestingly, we saw similar dynamic nuclear shape changes in the intravital movies of these tumor cells moving in vivo (Figure 5I, Movie S9). We quantified the nuclear aspect ratio (major axis/minor axis) and found that in both, in vivo and in 1D in vitro, the nuclear aspect ratio changes within a similar range (1.5–3.0). In contrast, nuclear aspect ratio in cells moving on 2D surface did not change over time (Figure 5J). These results are surprising, since unlike in an in vivo situation where it has been claimed that tumor cells change nuclear shape in order to pass through the narrow ECM pores, cells moving in 1D do not have any pore restriction. These results suggest that tumor cells have the intrinsic ability to modulate nuclear shape, while moving in 1D. Unlike fibroblasts, where a perinuclear actin cap and formin FMN2 have been proposed to regulate cell migration and nuclear shape [52,53,54], carcinoma cells used in this study do not rely on perinuclear actin cap or FMN2 for cell motility (Figures S4 and S5).

### 3.6. Downregulation of LINC Complex Proteins, SUN1 and SUN2, in Cancer Patients

Irregular nuclear shape and nuclear softness is seen in human laminopathies (a collection of human diseases including certain types of muscular dystrophies, dilated cardiomyopathy, and the premature aging disease Hutchinson–Gilford progeria syndrome) [55]. These diseases are primarily caused by the mislocalization or loss of nuclear envelope proteins. The Linker of Nucleoskeleton and Cytoskeleton, or LINC complex, comprising Nesprin and SUN family proteins of the nuclear envelope, performs a critical role of providing a physical link between the cell cytoskeleton and nucleus. Through this physical connection, LINC complex proteins regulate cellular mechanotransduction. Several studies have reported defects in cell motility after LINC complex disruption [56,57,58], however, the role of LINC complex in carcinoma cell motility in physiological environments is unknown.

**Figure 6:**
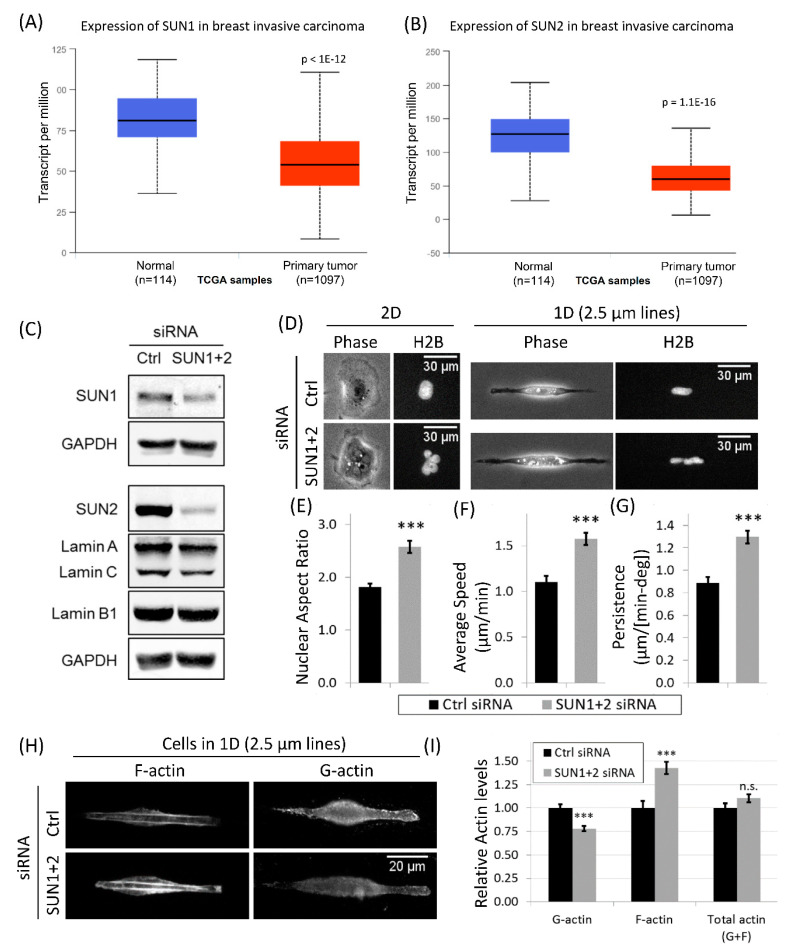
SUN1 and SUN2 downregulation in breast cancer patients; LINC complex disruption increases actin polymerization and tumor cell motility in 1D, but not in 2D. (**A**) Box plot analysis shows downregulation of SUN1 in primary breast tumor (*n* = 1097) versus normal breast tissue (*n* = 114) derived from RNA-Seq expression data from TCGA samples. (**B**) Box plot analysis shows downregulation of SUN2 in primary breast tumor (*n* = 1097) versus normal breast tissue (*n* = 114) derived from RNA-Seq expression data from TCGA samples. (**C**) Western blots showing SUN1 and SUN2 knockdowns in SUN1 siRNA + SUN2 siRNA treated tumor cells. Blots were also stained with Lamin A/C and Lamin B1 antibodies to check Lamin levels after SUN1 + 2 KD. (**D**) Images showing cellular (phase panels) and nuclear (GFP-H2B panels) morphologies after SUN1 + 2 KD in 1D (2.5 µm micropatterned line) and 2D. Note the multi-lobular nuclear morphology after SUN1 + 2 KD in both 1D and 2D. (**E**–**G**) Quantifications of nuclear aspect ratio (**E**), average tumor cell speed (**F**) and tumor cell persistence (**G**) after SUN1 + 2 KD in 1D. Data plotted as mean ± SEM, *n* = 22 cells (Ctrl siRNA) and 17 cells (SUN1 + 2 siRNA), *** *p* value < 0.001. (**H**) Images showing phalloidin (F-actin) and G-actin staining in control and SUN1 + 2 KD cells migrating in 1D. Images were acquired at the same exposure time and ND filter settings for each channel. (**I**) Quantifications of whole cell F-actin, G-actin, and total actin (F-actin + G-actin) levels in control and SUN1 + 2 KD cells migrating in 1D. Data normalized to Ctrl siRNA condition and shown as mean ± SEM, *n* = 32 cells (Ctrl siRNA) and 36 cells (SUN1 + 2 siRNA), *** *p* value < 0.001, n.s. not significant.

The LINC complex, Nesprin and SUN family proteins, reside in the outer and inner nuclear membranes, respectively [59,60,61]. Together they provide a physical link between the cellular cytoskeleton and nuclear lamina and have been implicated in nuclear mechano-transduction and cell motility [56,61,62]. Global loss of LINC complex components has previously been reported in breast cancer [63]. Analysis of The Cancer Genome Atlas (TCGA) dataset further confirmed that the expression of SUN1 and SUN2 is downregulated in breast cancer compared to normal breast tissue (Figure 6A,B). Moreover, downregulation of SUN1 and SUN2 was observed across tumor types (Figure S6A,B).

### 3.7. Disruption of LINC Complex Leads to Increased Tumor Cell Migration and Actin Polymerization in 1D

To further investigate the link between SUN proteins, nuclear shape and motility in carcinoma cells, we disrupted LINC complex by knocking down SUN proteins (SUN1 and SUN2). Using smartpool siRNAs for SUN1 and SUN2, we efficiently knocked down SUN1 and SUN2 (referred to SUN1 + 2 KD hereafter) in tumor cells (Figure 6C). Single cell immunofluorescence imaging confirmed depletion of SUN1 and SUN2 from the nuclear envelope after SUN1 + 2 KD (Figure S6C,D). We investigated nuclear morphology in SUN1 + 2 KD tumor cells, and found that cells showed irregular nuclear morphology with multiple lobes, folds and membrane invaginations in both 1D and 2D (Figure 6D, and Figure S6C–E, Movies S10 and S11), indicative of compromised nuclear mechanotransduction due to defects in nucleo-cytoskeleton integrity. Since Lamins have been shown to regulate nuclear envelope integrity [51,55], we checked Lamin levels after SUN1 + 2 KD, and found that Lamin A/C and Lamin B1 levels did not change much after SUN1 + 2 KD (Figure 6C). Single cell immunofluorescence imaging also confirmed no change in nuclear envelope localizations of Lamin A/C and Lamin B1 after SUN1 + 2 KD (Figure S6C–E), i.e., Lamins stay at the nuclear envelope even in the irregularly shaped nucleus after SUN1 + 2 KD. These results indicate that the nuclear morphology defects observed in SUN1 + 2 KD cells are not due to off-target effects, such as Lamins. Consistent with the nuclear phenotype, nuclear aspect ratio increased after SUN1 + 2 KD (Figure 6E).

Next, we investigated the motility parameters of tumor cells in 1D after SUN1 + 2 KD. Surprisingly, we found that both speed and persistence increased significantly after SUN1 + 2 KD (Figure 6F,G). This again suggests that carcinoma cell migration in 1D is linked to nuclear shape, as seen above (Figure 5). Since F-actin is the dominant cytoskeleton component regulating tumor cell migration [24,51], we investigated the changes in F-actin and G-actin (with specific antibody JLA20, see materials and methods and Figure S6F) levels in single tumor cells after SUN1 + 2 KD. We observed a significant increase in F-actin levels and a concomitant decrease in G-actin levels after SUN1 + 2 KD (Figure 6H,I) with no change in total actin levels after SUN1 + 2 KD (Figure 6I), indicating an upregulation in actin polymerization (G-actin to F-actin conversion). In cells moving on 2D substrates, no changes in tumor cell speed, persistence and actin polymerization (Figure S6G,H) were observed after SUN1 + 2 KD, indicating 1D-specific changes in cell motility and associated actin forces. Together, these results demonstrate that LINC complex disruption by SUN1 + 2 KD leads to 1D-specific increases in actin polymerization and tumor cell motility.

### 3.8. MKL1 Nuclear Translocation Regulates Increased Tumor Cell 1D Motility in SUN1 + 2 KD Cells

**Figure 7:**
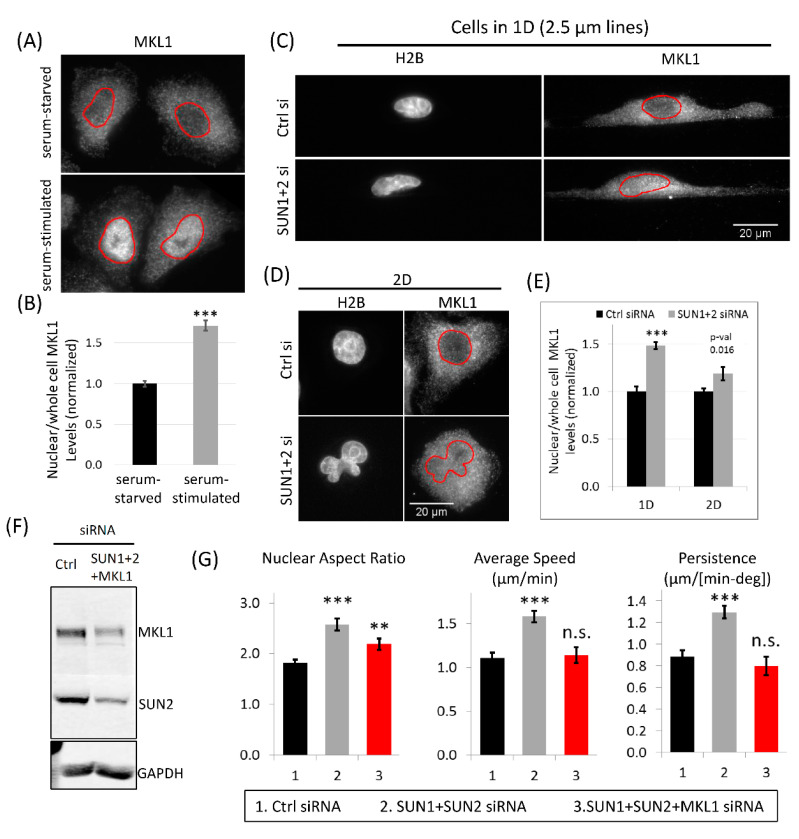
MKL1 is required for increased 1D tumor cell migration after SUN 1 + 2 KD. (**A**) MTLn3 carcinoma cells were stained with MKL1 antibody in serum-starved or serum stimulated conditions in 2D. Images show robust MKL1 nuclear translocation after serum stimulation (5% FBS for 5 min). Red outlines show nucleus boundary. (**B**) Quantification of nuclear/whole cell MKL1 levels in serum-starved or serum-stimulated MTLn3 carcinoma cells. Data normalized to steady state column and plotted as mean ± SEM, *n* = 30 cells (steady state condition) and 35 cells (serum stimulated condition), *** *p* value < 0.001. (**C**) Images of control siRNA or SUN1 + 2 siRNA treated H2B-MTLn3 cells migrating on 2.5 µm micropatterned 1D lines, stained with MKL1 antibody to show the nuclear MKL1 translocation after SUN1 + 2 KD. Red outlines show nucleus boundary. (**D**) Images of control siRNA or SUN1 + 2 siRNA treated H2B-MTLn3 cells migrating in 2D, stained with MKL1 antibody to show the nuclear MKL1 translocation after SUN1 + 2 KD. Red outlines show nucleus boundary. (**E**) Quantification of nuclear/whole cell MKL1 levels in control or SUN1 + 2 siRNA treated cells migrating in 1D or 2D. Data normalized to Ctrl siRNA columns and plotted as mean ± SEM, *n* = 15 cells (1D, Ctrl siRNA), 15 cells (1D, SUN1 + 2 siRNA), 37 cells (2D, Ctrl siRNA), and 24 cells (2D, SUN1 + 2 siRNA), *** *p* value < 0.001. (**F**) Western blots showing knockdowns of SUN2 and MKL1 in tumor cells treated with either control siRNA or SUN1 + SUN2 + MKL1 siRNAs. (**G**) Quantifications of nuclear aspect ratio, average tumor cell speed and tumor cell persistence after SUN1 + SUN2 + MKL1 KD in cells migrating in 1D. Data plotted as mean ± SEM, *n* = 20 cells for the SUN1 + SUN2 + MKL1 siRNA condition, ** *p*-value < 0.01, *** *p* value < 0.001, n.s. not significant. Control and SUN1 + 2 siRNA bars are shown here from Figure 6E–G for comparison.

We found decreased G-actin levels in SUN1 + 2 KD cells (Figure 6H,I). Myocardin-related transcription factor A (MRTF-A), also known as megakaryoblastic leukemia factor 1 (MKL1) shuttles to the nucleus from the cytoplasm in response to changes in G- and F-actin levels in the cell, where MKL1 binds serum-response factor (SRF) and initiates transcriptional programs for increased actin polymerization and cell motility [64,65]. In particular, transcription factor, MKL1 is known to translocate from the cytoplasm to the nucleus after reduced G-actin levels [66]. Therefore, we investigated if the MKL1-SRF signaling pathway plays any role in tumor cell motility in 1D. First, we checked if SRF-MKL1 signaling is relevant in our mammary tumor cells by looking at MKL1 distribution in mammary tumor cells before and after serum stimulation [66]. We stained serum-starved and serum-stimulated cells with MKL1 antibody. Compared to the cytosolic MKL1 distribution in serum-starved cells, we found a robust nuclear MKL1 localization in serum-stimulated cells (Figure 7A,B), indicating that MKL1-SRF pathway is active in mammary carcinoma MTLn3 cells. Since SUN1 + 2 KD led to a decrease in G-actin in cells moving in 1D (Figure 6H,I) and the drop in G-actin levels is known to cause MKL1 translocation from the cytosol to the nucleus [66], we hypothesized that SUN1 + 2 KD will lead to MKL1 nuclear translocation. We treated tumor cells with control or SUN1 + 2 siRNA and plated them on 1D or 2D substrates. MKL1 antibody staining of cells showed nuclear MKL1 translocation after SUN1 + 2 KD in both 1D and 2D. We quantified nuclear MKL1 signal and found that there was nearly 50% increase in nuclear MKL1 signal after SUN1 + 2 KD in cells moving in 1D. The increase in nuclear MKL1 signal in cells moving in 2D, though significant, was rather modest (~15%). These results indicate that loss of SUN proteins leads to nuclear MKL1 translocation, activating MKL1-SRF gene transcription machinery, leading to increased actin polymerization (Figure 6H,I) and increased tumor cell 1D motility (Figure 6F,G).

Next, we asked if MKL1 signaling is required for increased 1D motility of SUN1 + 2 KD cells. We treated tumor cell with either control siRNA or siRNAs for SUN1, SUN2 and MKL1. Western blot analysis showed nearly 80% KD of both MKL1 and SUN2 proteins (Figure 7F). We plated triple KD cells (SUN1 + 2 and MKL1) on 1D or 2D substrates and made time-lapse movies. We quantified nuclear aspect ratio, speed and persistence and found that MKL1 is required for high-speed 1D motility of SUN1 + 2 KD cells (Figure 7G). MKL1 KD, though, was not able to restore the nuclear morphology to what is seen in control cells (Figure 7G), indicating that MKL1 acts downstream of SUN1 + 2 mediated nuclear shape change. The effects of MKL1 on tumor cell motility were specific to 1D, as we did not see any change in tumor cell 2D motility after SUN1 + 2 and MKL1 KDs (Figure S6I).

## 4. Discussion

**Figure 8:**
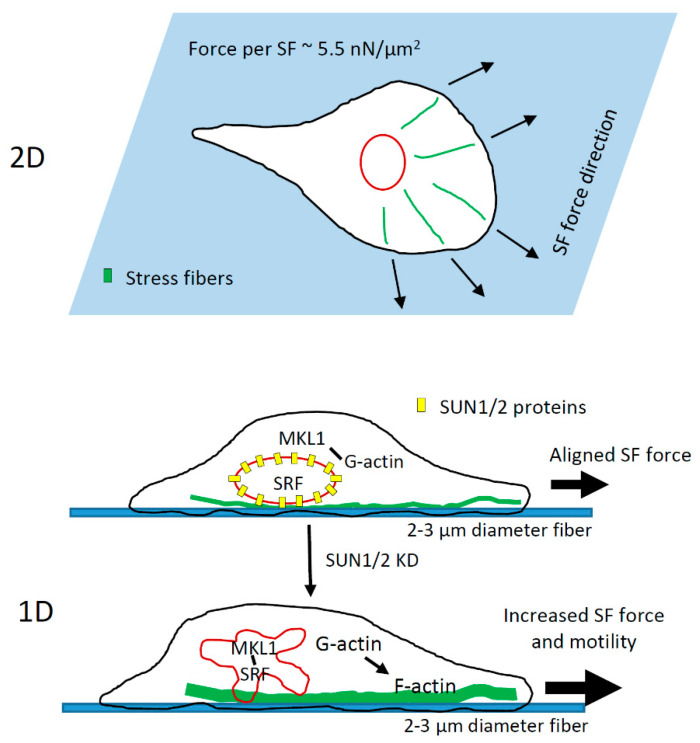
Model of tumor cell migration in fibrillar tumor microenvironment in vivo. During invasion, single tumor cells migrate along thin collagen fibers (2–3 µm in diameter) with high speeds. As opposed to 2D environment, where cell spreads leading to distributed actomyosin forces (force per stress fiber ~ 5.5 nN/µm^2^ [68,69]), in confined 1D environment, greater aligned actomyosin forces along the fiber 1D axis generate high-speed migration. This 1D high-speed migration is coupled to cell-intrinsic nuclear deformation even in the absence of any restricting ECM pores. Downregulation of LINC complex proteins (SUN1 and SUN2) in tumor cells leads to MKL1 nuclear translocation and increased SRF-MKL1 transcriptional activity, which upregulates genes for actin polymerization activity and further enhances tumor cell motility in 1D.

In this work, we evaluated the roles of cell-extrinsic (collagen fiber diameter) and cell-intrinsic (SUN and MKL1 signaling) factors influencing tumor cell motility in 1D, the fibrillar ECM microenvironment which tumor cells encounter in vivo to invade locally and migrate to the nearest blood vessel for hematogenous dissemination to secondary sites [5,15,67]. Our work demonstrates that tumor cells move the fastest on 2–3 µm diameter 1D fibers. 1D high-speed migration of tumor cells is closely linked to cell-intrinsic capacity for nuclear deformation. In addition, LINC complex disruption in tumor cells leads to even more enhancement in cell motility in 1D (Figure 8). Interestingly, these results were specific to 1D because tumor cells in 2D not only moved significantly slower, but also showed no changes in 2D motility after SUN and MKL1 signaling perturbations. Highly efficient high-speed tumor cell migration in 1D is achieved through bidirectional cell polarization in 1D and aligned cytoskeletal forces in the tumor cell along the ECM fiber axis, whereas in 2D, cytoskeletal forces are randomly distributed leading to lower net force in the direction of movement (Figure 8).

### 4.1. Collagen Fiber Diameter Is a Key Regulator of Tumor Cell Motility In Vivo

The tumor microenvironment ECM presents many biomechanical cues (ECM stiffness, crosslinking, topography, and alignment) to sustain high-speed tumor cell motility during tumor progression in vivo. In this study, we investigated the role of one such cue, the collagen fiber diameter, on tumor cell motility in vivo. Previous studies have imaged ECM collagen fibers in vivo in a variety of tissues (rat tail, ovarian tissue, mouse tumor xenografts, etc.) and reported their alignments [70,71,72]. A few studies have looked at collagen fiber diameters in healthy tissues and reported to be in the range of 500 nm to a few micrometers [73,74]. To our knowledge, we are the first to report collagen fiber diameter distribution in mammary tumors in vivo.

In this study, we reconstituted with high fidelity the high speed and persistence of streaming migration in tumor cells seen in vivo using synthetic fibers and micropatterned tracks composed of physiological ECM. The phenotypes of tumor cells moving on 1D fibers in vivo, including the formation of alternating patterns with macrophages and high speed persistent migration with pulsating nuclear deformation were exactly recreated in vitro and were fiber diameter dependent. Both micropatterned channels and fibers gave equivalent results and showed the same dependence of cell phenotype on the diameter of the linear ECM, indicating that the diameter and not curvature of the linear ECM dominated cell phenotype.

Previous studies utilizing 1D assays to model motility of different cell types (breast tumor, epithelial, fibroblast, myoblast) used 1D fiber diameters/line widths which were not thin (20–170 µm [75], 20 µm—2D [35]) or used a narrow range of diameters/widths (0.7–0.8 µm [17], 0.4–1.2 µm [76]). In general, these studies did not find changes in single cell speed as a function of varying 1D width. The Yamada group, on the other hand, used a range of micropatterned line widths (1–40 µm) to study fibroblast speed [38]. Interestingly, our results on the effect of varying 1D width (0.7–20 µm) on carcinoma cell speed are in close agreement with their fibroblast speed values, suggesting that extrinsic biomechanical cues from the microenvironment, rather than driver mutations and/or differentiated state of the cell, dominates motility phenotype in both normal and cancer cells.

### 4.2. Nuclear Shape Changes Are Linked to High-Speed Tumor Cell 1D Motility and Do Not Require ECM Pore Restriction

Consistent with previous 1D studies [52,77,78], we observed elongated nuclear morphology in tumor cells migrating in 1D (Figure 5G). Elongated nuclear morphology was further confirmed in carcinoma cells moving in vivo (Figure 5G). One mechanism of nuclear deformation is the compressive lateral actomyosin forces acting on both sides of the nucleus [78]. Accordingly, we saw alignments of F-actin and focal adhesions along the 1D axis (Figure 4). Moreover, inhibition of actomyosin contractility led to rounding of the nucleus in 1D (Figure 5C,F). Another mechanism for regulating nuclear shape is the apical F-actin caps [52,53]. Unlike fibroblasts, we did not see any apical F-actin caps in carcinoma cells (Figure S4), indicating that lateral actomyosin forces are the primary mechanism behind nuclear elongation in carcinoma cells in 1D.

Previous literature suggests that tumor cells deform the nucleus in order to squeeze through small ECM pores encountered during invasion and metastasis [51,79,80,81,82]. Here, we show that the tumor cell nucleus is inherently plastic and can display changes in shape in 1D, even in the absence of any restricting ECM pores. The amplitude of nuclear shape change observed in 1D was remarkably similar to nuclear shape changes observed in these cells in vivo (Figure 5H,I). These results suggest that rapid nuclear shape change is linked to high-speed tumor cell migration in 1D, since the inhibition of actomyosin contractility led to the disappearance of nuclear shape change cycles (nucleus became round) and greater than 50% reductions in cell motility parameters. Therefore, nuclear plasticity is not only required for passing through small pore openings in the ECM, but it is also linked to high-speed tumor cell migration in 1D, via the nucleo-cytoskeleton link.

Nuclear elongation is one of the characteristic features of migrating cells in vivo [51,79,83], which has been confirmed by many 1D studies [37,52,53,77,78,84]. The nucleus is the largest and stiffest organelle in the cell and it has been suggested to be an impediment for cell migration in vivo [79,82,85]. In contrast to the nucleus in normal cells, the nucleus in tumor cells is softer [86,87,88,89,90] and sometimes has aberrant morphology indicated by nuclear folds, invaginations, and blebs [91,92]. This allows tumor cells to modulate nuclear shape and escape through narrow openings frequently encountered during tumor cell invasion and metastasis.

### 4.3. Role of LINC Complex and MKL1-Driven Gene Transcription in High-Speed Tumor Cell Motility in 1D

The effect of LINC complex disruption on cell motility in 1D-like environments, to our knowledge, has not been previously reported. Using 2D substrates, previous studies [56,57,58] have reported reductions in cell motility after LINC complex disruption in fibroblast and endothelial cells. However, in the tumor cells studied here, we did not see any changes in 2D cell motility after SUN1 + 2 knockdown (Figure S6G). Interestingly, the same tumor cells showed 50% higher motility after SUN1 + 2 knockdown when moving in 1D environment (Figure 6F). Previous studies have shown that disrupting nuclear lamins lead to nuclear softening [93,94]. In SUN1 + 2 knockdown tumor cells, we saw irregular shaped nuclei with nuclear envelope folds, invaginations, and blebs (Figure 6D, Movies S10 and S11). These irregular nuclear features are often seen in cancer cells from patients and in normal cells with reduced Lamin protein expression [51,55]. We therefore speculate that SUN1 + 2 knockdown leads to a softer nucleus in carcinoma cells, allowing them to squeeze through tight spaces encountered during invasion and metastasis. The increased nuclear flexibility of SUN1 + 2 KD carcinoma cells to pass through narrow ECM constrictions combined with their increased motility in 1D make these cells super invaders during mammary tumor invasion. Two previous studies, one utilizing a 3D transwell migration assay [85] and the other microfluidics [81], which mimic in vivo environment more closely than 2D assay, support our findings—that the nucleus is an impediment to cell migration in 3D/1D like environments and that softening the nucleus leads to higher cell motility.

Forces required for increased motility of SUN1 + 2 knockdown cells are generated by increased F-actin polymerization (Figure 6H,I), suggesting upregulation of genes involved in F-actin polymerization. Changes in G- and F-actin levels in cells are known to cause nuclear translocation of MKL1 [66]. The activation of the SRF-MKL pathway upon nuclear translocation targets the transcription of many genes involved in the regulation of actin polymerization, dynamics and protrusion (e.g., cofilin, WASP, Arp2/3), cell–cell and cell–ECM adhesion (e.g., integrin, vinculin and cadherin), enhancing cell motility through a positive feedback regulation of actin polymerization [64,65,95]. The role of MKL1 in cell motility is already known from many previous studies. MKL1 depletion leads to defects in cell motility in multiple cells including breast cancer cells [95,96,97]. Our novel observation that LINC complex disruption leads to increased MKL1 nuclear translocation suggests crosstalk between LINC complex and SRF-MKL pathway and an important role of SRF-MKL driven gene transcription in 1D tumor cell motility. In fact, using triple knockdowns (SUN1, SUN2, and MKL1) in tumor cells, we show that MKL1 is required for enhanced 1D motility of SUN1 + 2 KD cells (Figure 7F,G). Future studies are required to identify specific SRF-MKL transcriptional genes involved in tumor cell motility in physiological environments.

## Supplementary Materials

The following are available online at https://www.mdpi.com/article/10.3390/cells10061549/s1.
