# Supplementary material for: SUN-MKL1 Crosstalk Regulates Nuclear Deformation and Fast Motility of Breast Carcinoma Cells in Fibrillar ECM Microenvironment
Source: Cells. 2021 Jun 19;10(6):1549. doi: 10.3390/cells10061549 (PMC8234170; doi:10.3390/cells10061549)
Supplement: Supplementary file 1 [file cells-10-01549-s001.zip › Supplementary materials -final/cells-1274563 supplementary materials final.pdf]

Supplementary Materials

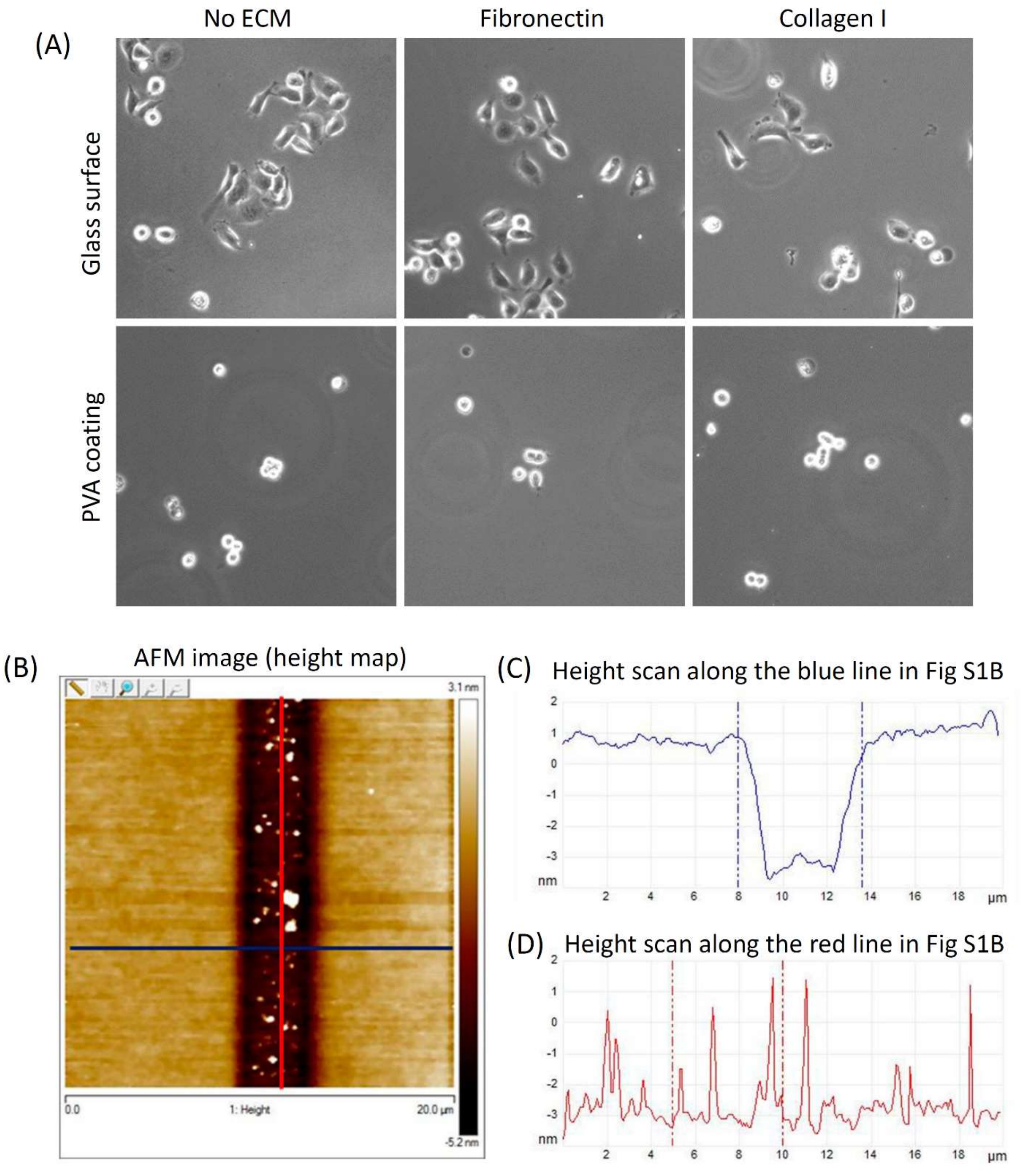

Figure S1. Surface characterization in 1D assays. (A) Tumor cells do not adhere and spread on PVA-coated surface. Images of MTLn3 cells plated on either glass or PVA-coated surface with and without ECM (fibronectin or collagen) coating. (B) The dimensions of 1D micro-patterned lines were evaluated using AFM. Image shows the height map of a 1D line and the surrounding area. (C) Height scan along the blue line in figure S1(B) shows that the thickness of 1D lines is approximately 2.5 µm. (D) Height scan along the red line in figure S1(B) shows that the depth of 1D lines is approximately 3-4 nm.

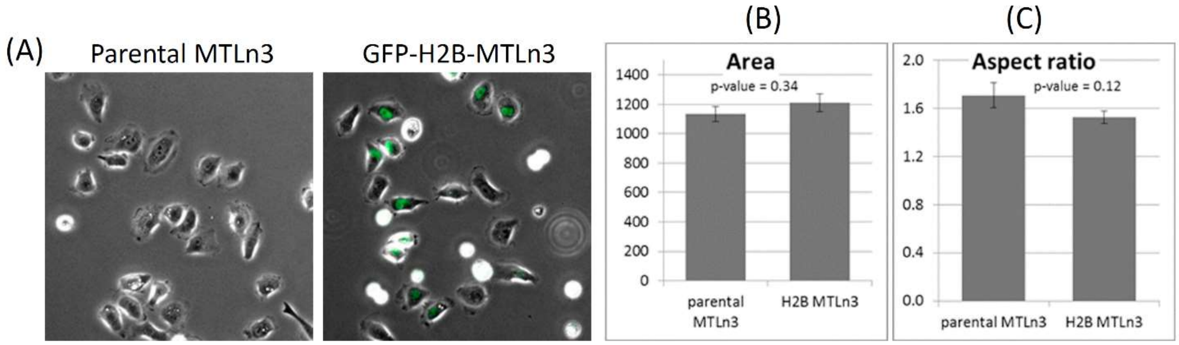

Figure S2. Cellular features of stable GFP-H2B-MTLn3 cells are similar to parental MTLn3 cells. (A) Parental or GFP-H2B expressing MTLn3 cells were plated on ECM coated 2D glass surfaces. Phase and phase+GFP channel images were acquired for the parental and GFP-H2B MTLn3 cells, respectively. (B, C) Quantifications of cell spread area (B) and cellular aspect ratio (C) in parental and GFP-H2B expressing MTLn3 cells. Data plotted as mean ± SEM, N = 40 parental MTLn3 cells and 34 GFP-H2B MTLn3 cells.

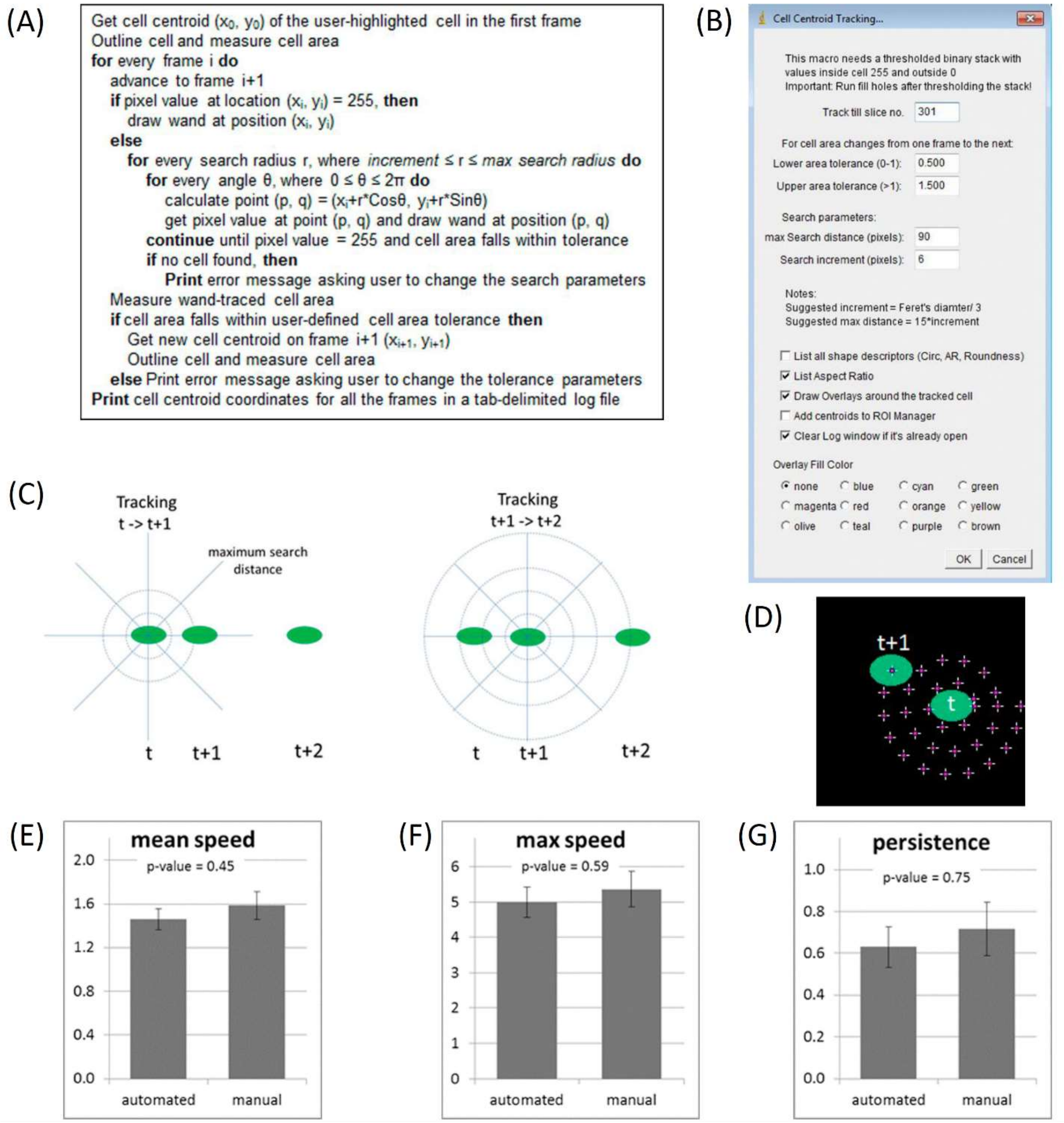

Figure S3. Description of "Cell centroid tracking" macro (included with the article) for automated tracking of single tumor cells. (A) Algorithm for cell centroid tracking macro. (B) Macro user-interface (UI) showing different parameters (e.g. maximum search distance, search increment etc.) which user can adjust for accurate cell tracking. (C) Schematic of cell nucleus tracking from time, t to t+1 and t+1 to t+2. Green ovals represent cell nucleus at different times and circles of different radii represent successive search increments. (D) An example of cell nucleus tracking from time, t to t+1. Green ovals represent cell nucleus at different time points and cross-hairs show all the points generated by the macro in search of the cell nucleus at time t+1. Search takes place in a clockwise manner. (E, F, G) GFP-H2B expressing tumor cells moving on 2 μm fibers were tracked using automated method (with Cell centroid tracking macro) and by marking cell centroid manually in each frame of the movie. Data plotted as mean ± SEM, N = 6 cells each for the automated and manual analysis. Bar plots for the cell migration parameters - average speed (E), maximum speed (F) and persistence (G), show that the automated method is as accurate as the manual method. Therefore, all subsequent single tumor cell speed and persistence calculations were performed using automated Cell centroid tracking macro.

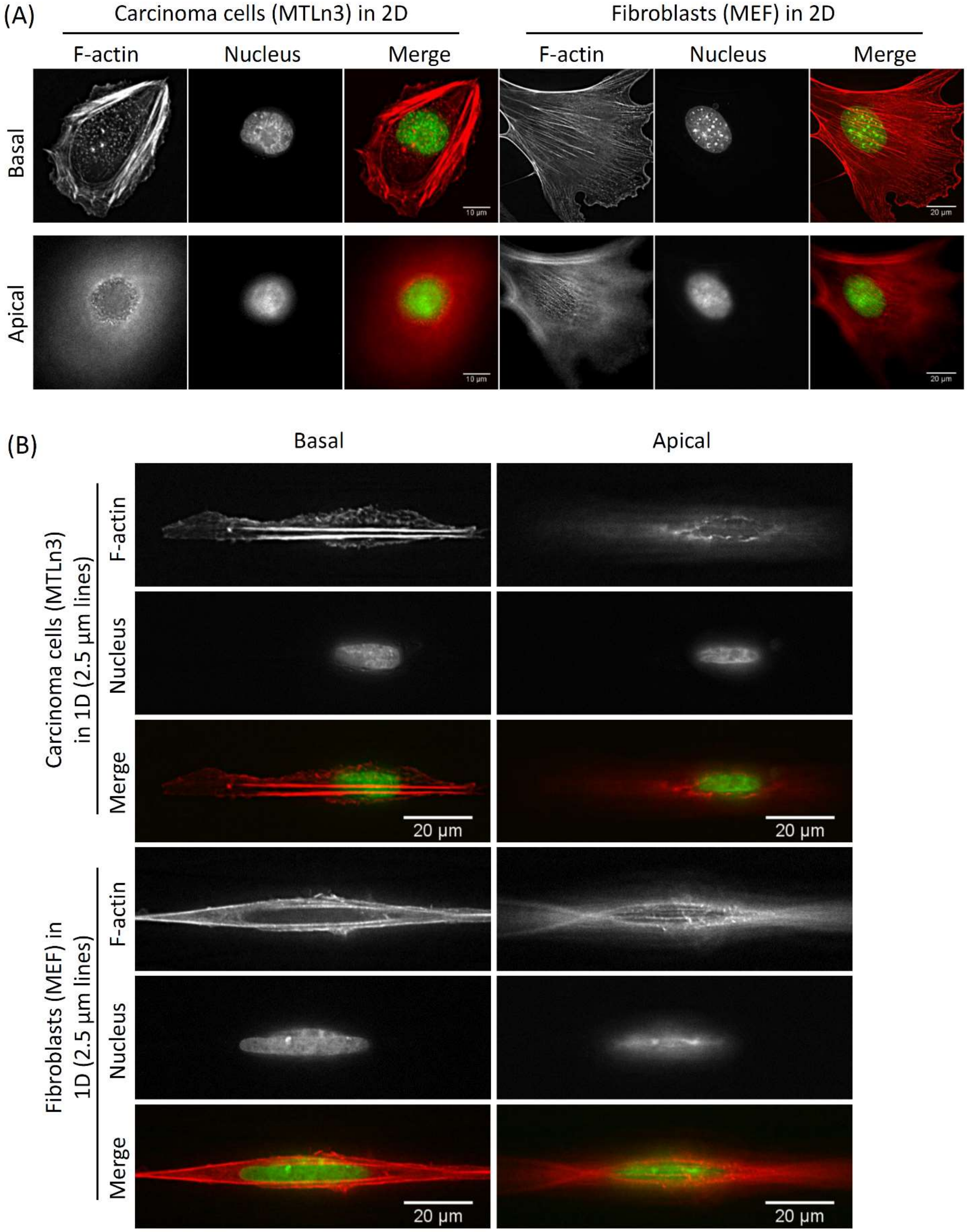

Figure S4. MTLn3 tumor cells do not display actin nuclear cap in 2D or 1D. (A) MTLn3 tumor cells and MEF fibroblasts were plated on 2D substrate and stained with phalloidin and DAPI. Top panels show basal plane and the bottom panels show apical plane from the z-stack. Note that MEFs have apical F-actin fibers which are missing in the MTLn3 cell. (B) MTLn3 tumor cells and MEF fibroblasts were plated on 1D substrate (2.5 μm micropatterned line) and stained with phalloidin and DAPI. Left panels show basal plane and the right panels show apical plane from the z-stack. Note that MEFs have apical F-actin fibers which are missing in the MTLn3 cell.

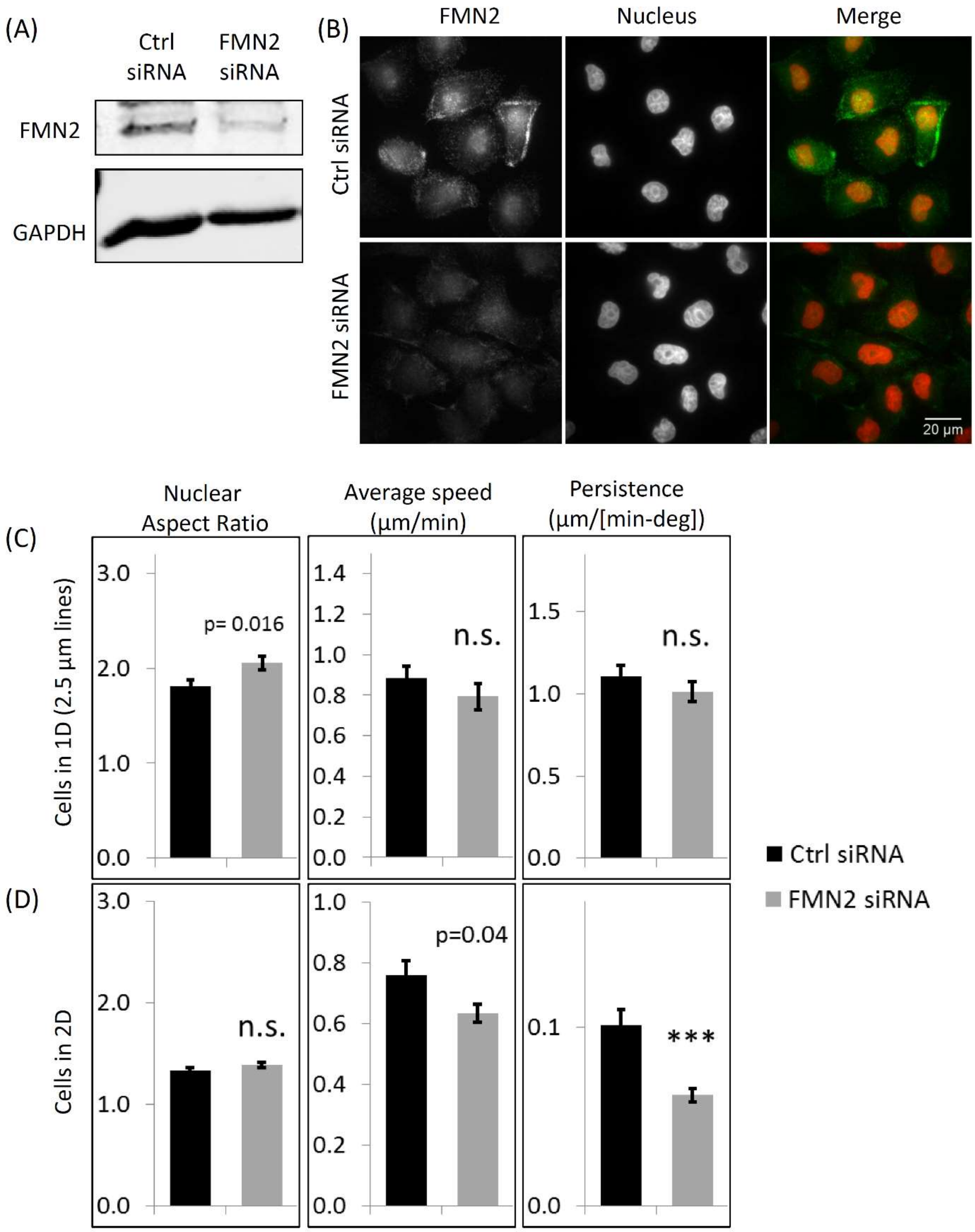

Figure S5. Formin FMN2 does not play any role in tumor cell motility in 1D. (A) Western blot showing FMN2 KD with FMN2 smartpool siRNA for 48 hour. (B) Immunofluorescence images of MTLn3 cells treated with control or FMN2 smartpool siRNA for 48 hour were stained with anti-FMN2 antibody. Please note the decrease in FMN2 fluorescence signal indicating efficient FMN2 KD. (C, D) Quantifications of nuclear aspect ratio, speed and persistence in cells moving in 1D (C) or 2D (D). Data plotted as mean ± SEM, N = 22 cells (1D, Ctrl siRNA), 31 cells (1D, FMN2 siRNA), 25 cells (2D, Ctrl siRNA), 28 cells (2D, FMN2 siRNA).

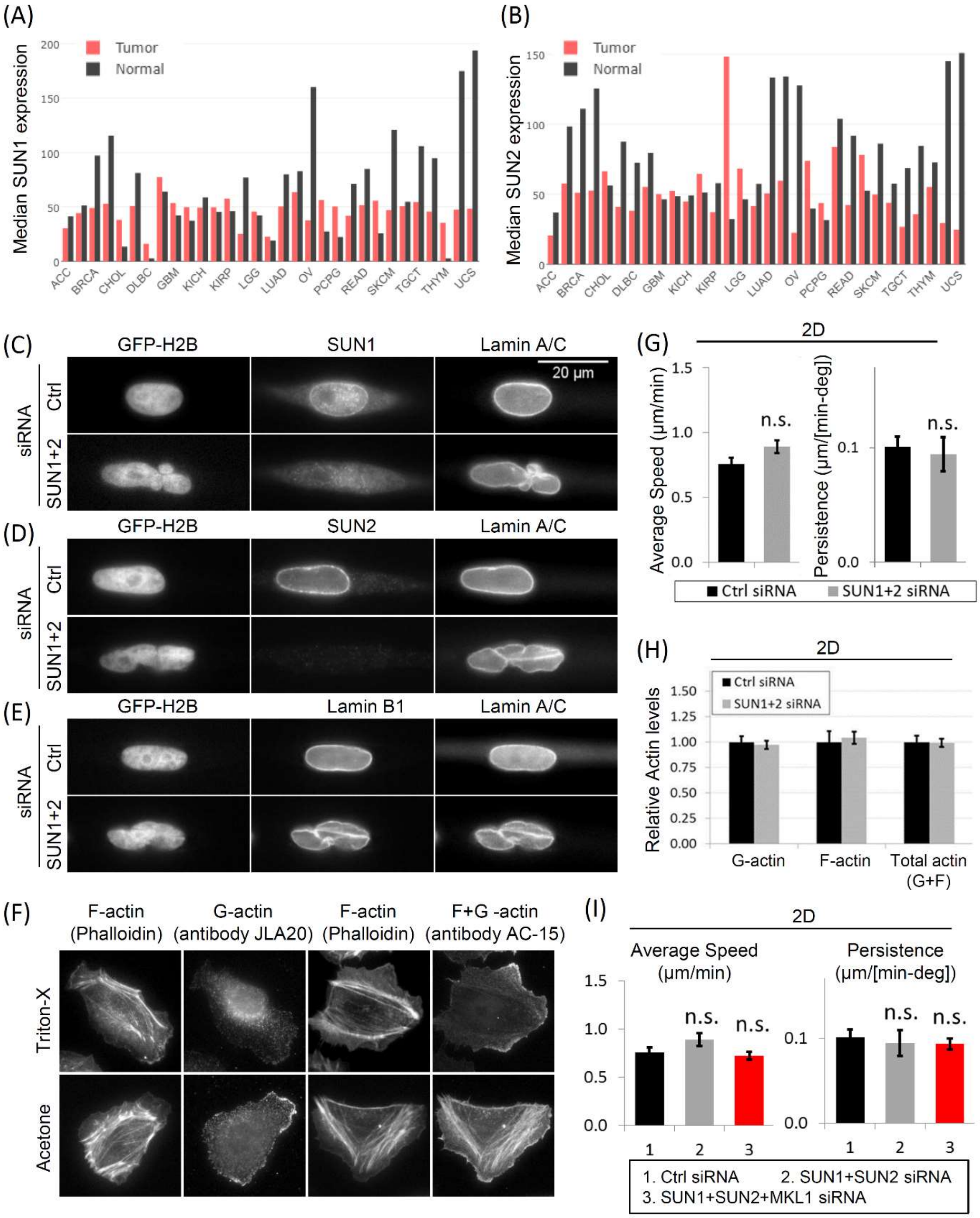

Scheme 6. SUN1, SUN2 and MKL1 do not play any role in 2D tumor cell motility. (A, B) RNA-Seq data analysis for median SUN1 and SUN2 expression in tumor vs normal tissue across tumor types from the TCGA and GTEx datasets. Tumor type abbreviations - ACC: Adrenocortical carcinoma, BLCA: Bladder Urothelial Carcinoma, BRCA: Breast invasive carcinoma, CESC: Cervical squamous cell carcinoma and endocervical adenocarcinoma, CHOL: Cholangio carcinoma, COAD: Colon adenocarcinoma, DLBC: Lymphoid Neoplasm Diffuse Large B-cell Lymphoma, ESCA: Esophageal carcinoma, GBM: Glioblastoma multiforme, HNSC: Head and Neck squamous cell carcinoma, KICH: Kidney Chromophobe, KIRC: Kidney renal clear cell carcinoma, KIRP: Kidney renal papillary cell carcinoma, LAML: Acute Myeloid Leukemia, LGG: Brain Lower Grade Glioma, LIHC: Liver hepatocellular carcinoma, LUAD: Lung adenocarcinoma, LUSC: Lung squamous cell carcinoma, MESO: Mesothelioma, OV: Ovarian serous cystadenocarcinoma, PAAD: Pancreatic adenocarcinoma, PCPG: Pheochromocytoma and Paraganglioma, PRAD: Prostate adenocarcinoma, READ: Rectum adenocarcinoma, SARC: Sarcoma, SKCM: Skin Cutaneous Melanoma, STAD: Stomach adenocarcinoma, TGCT: Testicular Germ Cell Tumors, THCA: Thyroid carcinoma, THYM: Thymoma, UCEC: Uterine Corpus Endometrial Carcinoma, UCS: Uterine Carcinosarcoma, UVM: Uveal Melanoma. (A) Control and SUN1+2 siRNA treated tumor cells were stained with SUN1 and Lamin A/C

antibodies. Immunofluorescence images show multi-lobular nucleus with no SUN1 localization at the nuclear envelope, whereas Lamin A/C localization was unchanged after SUN1+2 KD. (B) Control and SUN1+2 siRNA treated tumor cells were stained with SUN2 and Lamin A/C antibodies. Immunofluorescence images show multi-lobular nucleus with no SUN2 localization at the nuclear envelope, whereas Lamin A/C localization was unchanged after SUN1+2 KD. (C) Control and SUN1+2 siRNA treated tumor cells were stained with Lamin B1 and Lamin A/C antibodies. Immunofluorescence images show multi-lobular nucleus with no changes in Lamin B1 and Lamin A/C localizations after SUN1+2 KD. (D) Staining of tumor cells in 2D with phalloidin and either JLA antibody (specific for G-actin), or AC-15 antibody (labels both G- and F-actin) under Triton-X or acetone permeabilization conditions. Images show that acetone, which denatures the proteins enable better localizations of G-actin and F-actin in the cells as compared to Triton-X. (E) Quantifications of average tumor cell speed and persistence in control and SUN1+2 KD cells migrating in 2D. Data plotted as mean ± SEM, N=25 cells (Ctrl siRNA) and 22 cells (SUN1+2 siRNA). (F) Quantifications of whole cell F-actin, G-actin and total actin (F-actin + G-actin) levels in control and SUN1+2 KD cells migrating in 2D. Data normalized to Ctrl siRNA condition and plotted as mean ± SEM, N=23 cells (Ctrl siRNA) and 27 cells (SUN1+2 siRNA). (G) Quantifications of average tumor cell speed and tumor cell persistence after SUN1+SUN2+MKL1 KD in cells migrating in 2D. Data plotted as mean ± SEM, N=22 cells for the SUN1+SUN2+MKL1 siRNA condition. Control and SUN1+2 siRNA bars are shown here from figure S6G for comparison.

Supplemental Movies:

Movie 1: 3D reconstruction movie of collagen fibers *in vivo*, imaged using second harmonic generation (SHG) intravital multiphoton microscopyin MTLn3 tumor model.

Movie 2: Movie showing tumor cells (GFP-H2B expressing MTLn3) moving on ECM coated 1D fibers. Upper panel shows phase contrast channel and lower panel shows GFP channel. Time in hour: minute.

Movie 3: Movie showing tumor cell (MTLn3) and macrophage (BAC) pairing and alternating streaming pattern on 1D fibers. Time in hour: minute.

Movie 4: Left: tumor cells moving in 2D. Right: tumor cells moving on 2.5 μm micro-patterned 1D lines. Upper panels show phase contrast channel. Lower panels show tracking of individual tumor cells based on GFP-H2B thresholding and automated tracking using Cell centroid tracking macro (Figure S3). Different colors mark nuclei of different single tumor cells. Time in hour: minute.

Movie 5: CellLight Talin-GFP expressing MTLn3 tumor cells moving on ECM coated 1D fiber. Time in hour: minute.

Movie 6: GFP-H2B expressing MTLn3 tumor cells moving on ECM coated 1D fibers, before and after 5 μM blebbistatin treatment. Time in hour: minute.

Movie 7: GFP-H2B expressing MTLn3 tumor cells moving on ECM coated 1D fiber. Time in hour: minute.

Movie 8: GFP-H2B expressing MTLn3 tumor cells moving on ECM coated 2D surface. Time in hour: minute.

Movie 9: Intravital imaging of GFP-H2B expressing MTLn3 tumor cells moving *in vivo*.

Movie 10: GFP-H2B expressing MTLn3 tumor cells treated with SUN1+2 siRNA were plated on 2D substrate and time-lapse images for phase and GFP-H2B channels were recorded every 2 min. Time in hour: minute.

Movie 11: GFP-H2B expressing MTLn3 tumor cells treated with SUN1+2 siRNA were plated on 2.5 μm, 1D micro-patterned lines and time-lapse images for phase and GFP-H2B channels were recorded every 2 min. Time in hour: minute.
