# Supplementary figures and images for: SUN-MKL1 Crosstalk Regulates Nuclear Deformation and Fast Motility of Breast Carcinoma Cells in Fibrillar ECM Microenvironment
Source: Cells. 2021 Jun 19;10(6):1549. doi: 10.3390/cells10061549 (PMC8234170; doi:10.3390/cells10061549)

# Full blot images for Figure 6(C)

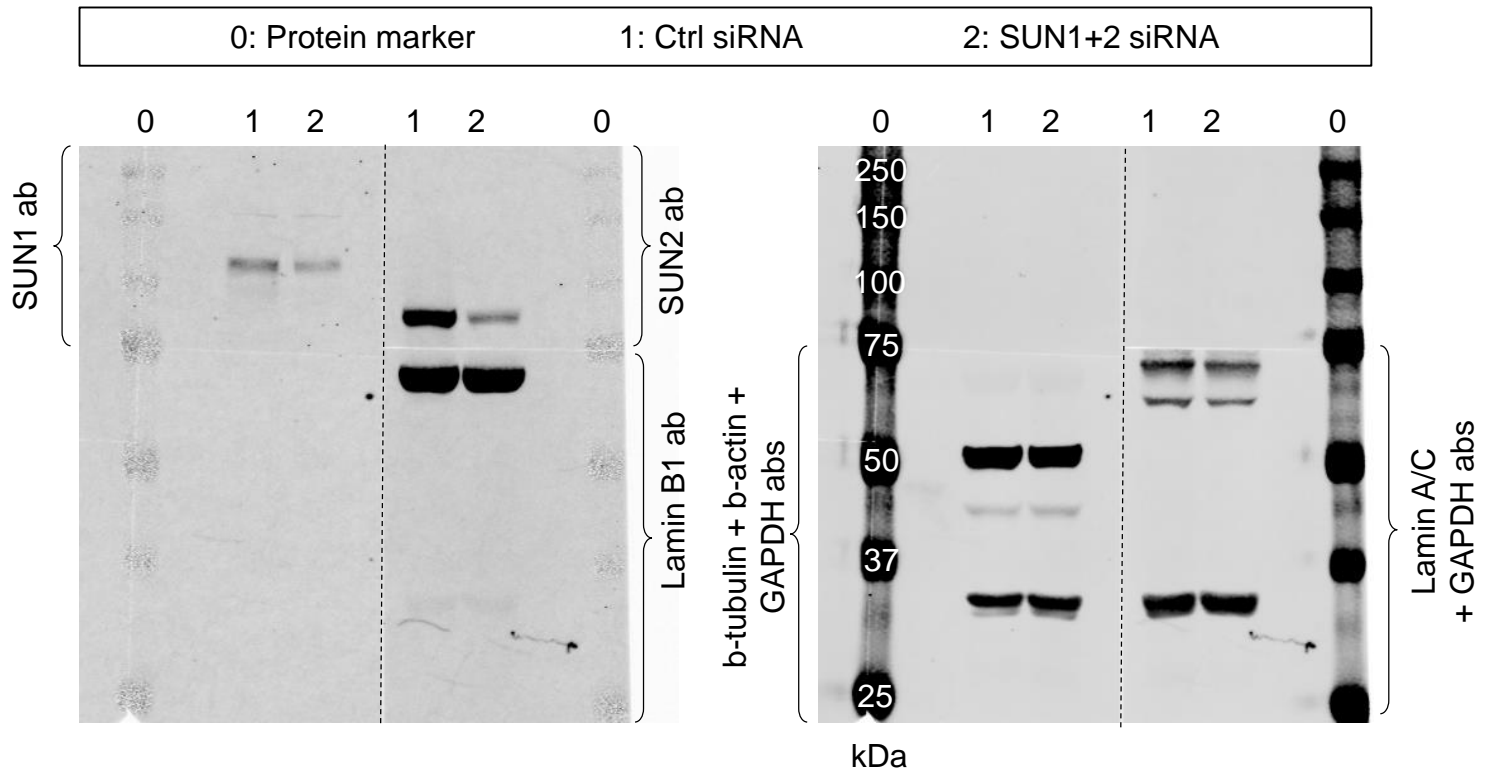

## Full blot images for Figure 7(F)

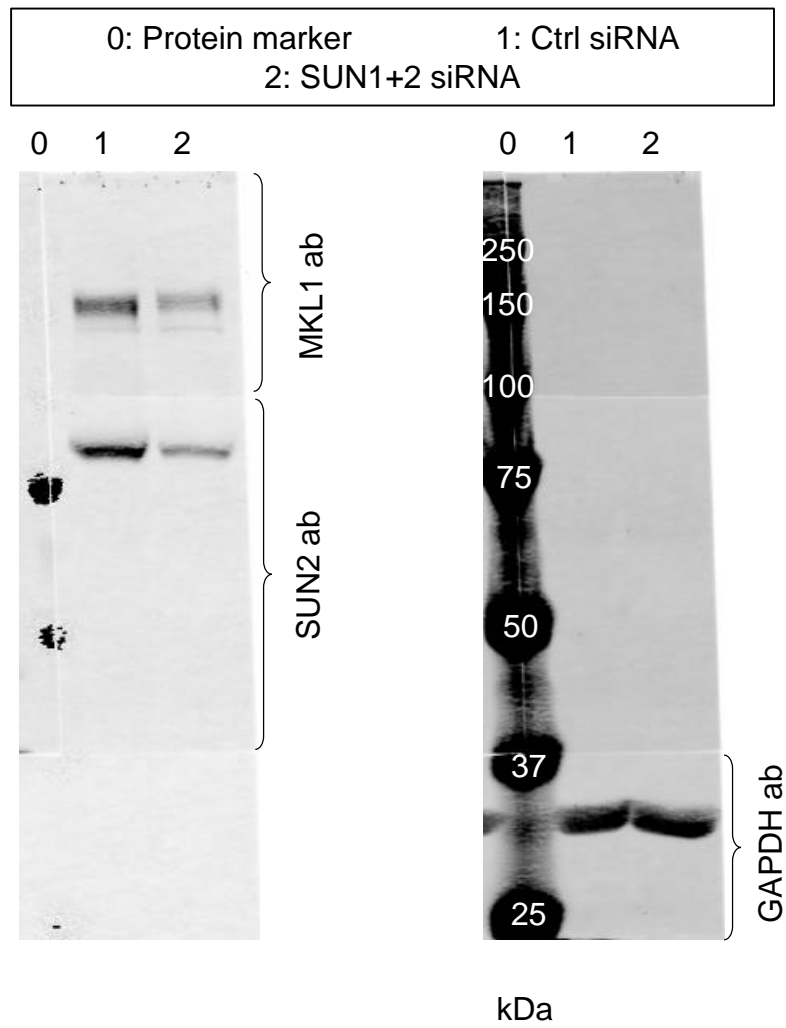

# Full blot images for Figure S5(A)

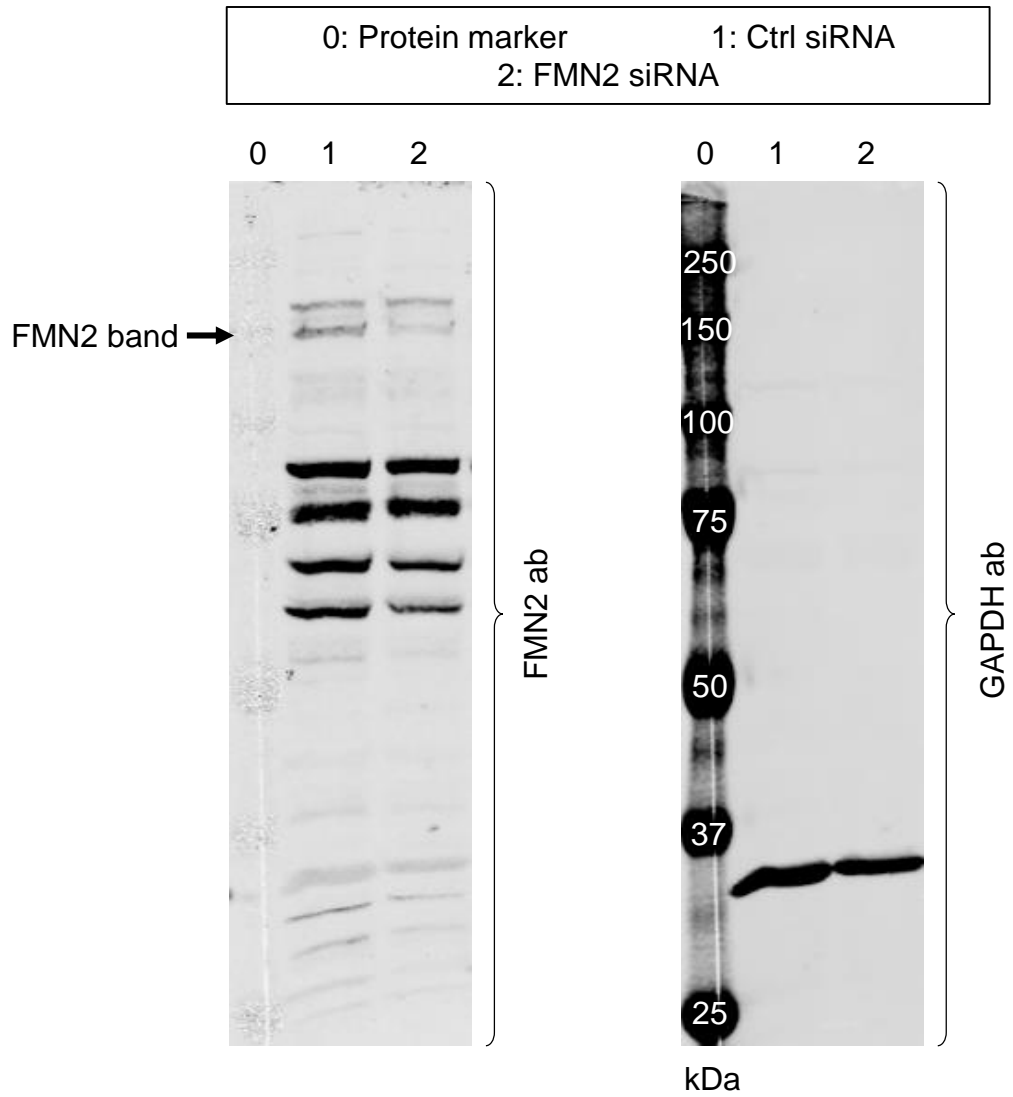

Supplement: Supplementary file 1 [file cells-10-01549-s001.zip › Supplementary materials -final/WB_Full blot images.pdf]
